# QSAR Assessing the Efficiency of Antioxidants in the Termination of Radical-Chain Oxidation Processes of Organic Compounds

**DOI:** 10.3390/molecules26020421

**Published:** 2021-01-14

**Authors:** Veronika Khairullina, Irina Safarova, Gulnaz Sharipova, Yuliya Martynova, Anatoly Gerchikov

**Affiliations:** Faculty of Chemistry, Bashkir State University, 450076 Ufa, Russia; safarova-77@mail.ru (I.S.); gulnaz-sharipova@list.ru (G.S.); Martynovayuz@gmail.com (Y.M.); gerchikov@inbox.ru (A.G.)

**Keywords:** antioxidant activity, antioxidants, QSAR models, GUSAR 2013 program, QNA descriptors, MNA descriptors

## Abstract

Using the GUSAR 2013 program, the quantitative structure–antioxidant activity relationship has been studied for 74 phenols, aminophenols, aromatic amines and uracils having lgk_7_ = 0.01–6.65 (where k_7_ is the rate constant for the reaction of antioxidants with peroxyl radicals generated upon oxidation). Based on the atomic descriptors (Quantitative Neighborhood of Atoms (QNA) and Multilevel Neighborhoods of Atoms (MNA)) and molecular (topological length, topological volume and lipophilicity) descriptors, we have developed 9 statistically significant QSAR consensus models that demonstrate high accuracy in predicting the lgk_7_ values for the compounds of training sets and appropriately predict lgk_7_ for the test samples. Moderate predictive power of these models is demonstrated using metrics of two categories: (1) based on the determination coefficients R^2^ (R^2^_TSi_, R^2^_0_, Q^2^(_F1_), Q^2^(_F2_), $\bar{R_{\mathrm{mTSi}}^{2}}$) and based on the concordance correlation coefficient (CCC)); or (2) based on the prediction lgk_7_ errors (root mean square error (RMSEP), mean absolute error (MAE) and standard deviation (S.D.)) The RBF-SCR method has been used for selecting the descriptors. Our theoretical prognosis of the lgk_7_ for 8-PPDA, a known antioxidant, based on the consensus models well agrees with the experimental value measure in the present work. Thus, the algorithms for calculating the descriptors implemented in the GUSAR 2013 program allow simulating kinetic parameters of the reactions underling the liquid-phase oxidation of hydrocarbons.

## 1. Introduction

Currently, the antioxidant properties of natural and synthetic substances are in the focus of chemical, biological, and pharmacological studies. This diversified interest relates to the possible use of these compounds as stabilizers in food, polymer, and fuel industries. Additionally, antioxidants are promising for anticancer and antitumor pharmaceuticals [1,2,3,4,5,6,7,8,9,10,11,12,13]. Indeed, antioxidants are able to deactivate both typical radical intermediate products and molecular oxidation products, the excessive formation of which can provoke the appearance and development of various pathologies, including cancer, premature aging, diabetes, etc. [13,14,15,16,17,18,19,20].

As is known, oxidation of organic compounds occurs via the chain mechanism that involves alkyl and peroxyl radicals [14,15]. The accumulated organic hydroperoxides decompose generating radicals, thereby increasing the rate of oxidation. The oxidation can be inhibited by the following three ways:

- Termination of the chains by the reaction of the inhibitor with peroxyl radicals;
- Termination of the chains by the reaction of the inhibitor with alkyl radicals;
- Termination with the compounds inducing the non-radical decomposition of the organic hydroperoxide (only if the latter is the main auto-initiator).

According to Denisov et al. [14], antioxidants are divided in 7 groups depending on the deactivation regime of oxidation. A detailed analysis of each of them is presented in the Supplementary Materials.

A single criterion for evaluating for assessing the antioxidant activity (AOA) of organic compounds is absent. AOA can be characterized with various semi-quantitative and quantitative parameters. The IC_50_ concentration is widely used as a semi-quantitative measure of AOA (IC_50_ relates to the intensity of the oxidation process reduced two times or the amount of malonic dialdehyde formed upon the oxidation of unsaturated fatty acids [21,22,23,24,25,26]). The reliable quantitative characterization of AOA may be based on the rate constants of the reaction of antioxidant molecule with radicals relaying the oxidation chain. These rate constants are usually designated as k_7_ or k_In_ in the case of compositions based on different antioxidants. Measuring these kinetic parameters may be performed by known methods of chemical kinetics [14,27].

In the scientific literature, one can find the works on QSAR modeling of antioxidants from the classes of phenols and polyphenols, in which the descriptors were calculated using quantum chemistry methods. In these studies, indicative variables were used jointly with quantum-chemically calculated descriptors (energies of the frontier molecular orbitals E_HOMO_ and E_LUMO_, difference in the heats of formation of phenol and its radical (ΔΔH_R_), bond dissociation enthalpies etc.) [28,29,30,31,32,33,34]. However, all these works are built on fairly narrow sets of similar compounds, e.g., the effect of para-substituents on the antioxidant activity of 10 and 27 phenol derivatives was studied in works [28] and [29], respectively. Based on quantum chemical calculations, the authors of [28] found that the smaller the BDE number, the higher the antioxidant activity of the modeled compounds. In work [29], using the model reaction of AIBN-induced styrene oxidation (T = 323 K), it was found that the inhibition rate constants k_7_ for para-substituted phenols correlates with the Brown-Okamoto constants σ_p_^+^. The smaller its numerical value for the para-substituent (i.e., the higher the positive inductive effect of this substituent, the higher the numerical value of k_7_). In the work [30], as a result of modeling 14 para-substituted phenols, 4 QSAR models were built, in which the reducing potency (E_mid_) correlates with quantum chemical descriptors such as ionization potential of the parent molecule (IP_p_), spin delocalization of the intermediate radical cation (D_s_^c^), LUMO of the parent molecule (E_LUMOp_), difference between the heats of formation of the phenoxyl radical (ΔΔH_R_) and parent molecule (ΔΔH_p_). In work [31] it was shown that quantum chemical descriptors such as ionization potentials (IP), absolute electronegativity (χ), activation energy (ΔΔH^#^), difference in the heats of formation of compounds and their radicals (ΔΔH_R_) make a decisive contribution to the antioxidant activity of phenols. The calculation of these descriptors was performed using the AM1 method (MOPAC 6.0 software). In work [32], moderate correlations for 30 Schiff bases were obtained between antiradical activity and quantum chemistry descriptors including the bond dissociation enthalpies related to the first and second hydrogen atom transfer (BDE and BDEd), the number of OH groups (nOH); the spin density of the active OH groups (SD); and the free enthalpy of the reaction of the reactivity of phenolic Schiff bases with the DPPH radical (ΔG). Work [33] reported that the antioxidant activity of phenols correlates with electron affinity (EA) and hardness (η) calculated with AM1 and PM3. The authors of work [34] based on 15 antioxidants built a QSAR model with high values of statistical parameters using enthalpy of homolytic dissociation of OH bonds (BDE-OH) and ionization potential (IP), and two lipophilic parameters, lipophilicity (LogP) and relative lipophilicity (LogD). In this model, the most pronounced antioxidant effect was found for the compounds with electron-donor groups directly bonded with the aromatic ring. The results of these and other similar studies are fundamental. However, it is incorrect to perform virtual screening with QSAR models based on training samples with less than 30 compounds. Thus, the use of these quantum-chemically calculated descriptors and values of Taft constants, despite of their clear physical meaning, is difficult for wide sets of compounds with different structures, i.e., such approaches are applicable only to training sets of closely related compounds.

To modelling the large-sized training sets of the diverse compounds, one has to use the methods for calculating descriptors that allow rationalizing the QSAR model development process. GUSAR 2013 (General Unrestricted Structure Activity Relationships) is one of the programs for calculating physicochemical and structural descriptors, selection of the most significant of them and developing a QSAR consensus model based on them [35,36,37,38]. This program for the formation of independent descriptors and their selection at the stage of the formation of regression models uses unique approaches, which we describe in detail in Supplementary Materials. The program has demonstrated its efficiency for modeling various types of biological activity [35,36,37,38,39,40,41,42,43,44,45,46]. It is important to note that GUSAR 2013 allows prompt processing (in a matter of seconds) various structural and physicochemical descriptors, automatic selecting the most significant of them, and develop statistically significant QSAR consensus models. As far as we can judge, GUSAR 2013 was not used for research on the antioxidant activity of QSAR.

**Figure 1:**
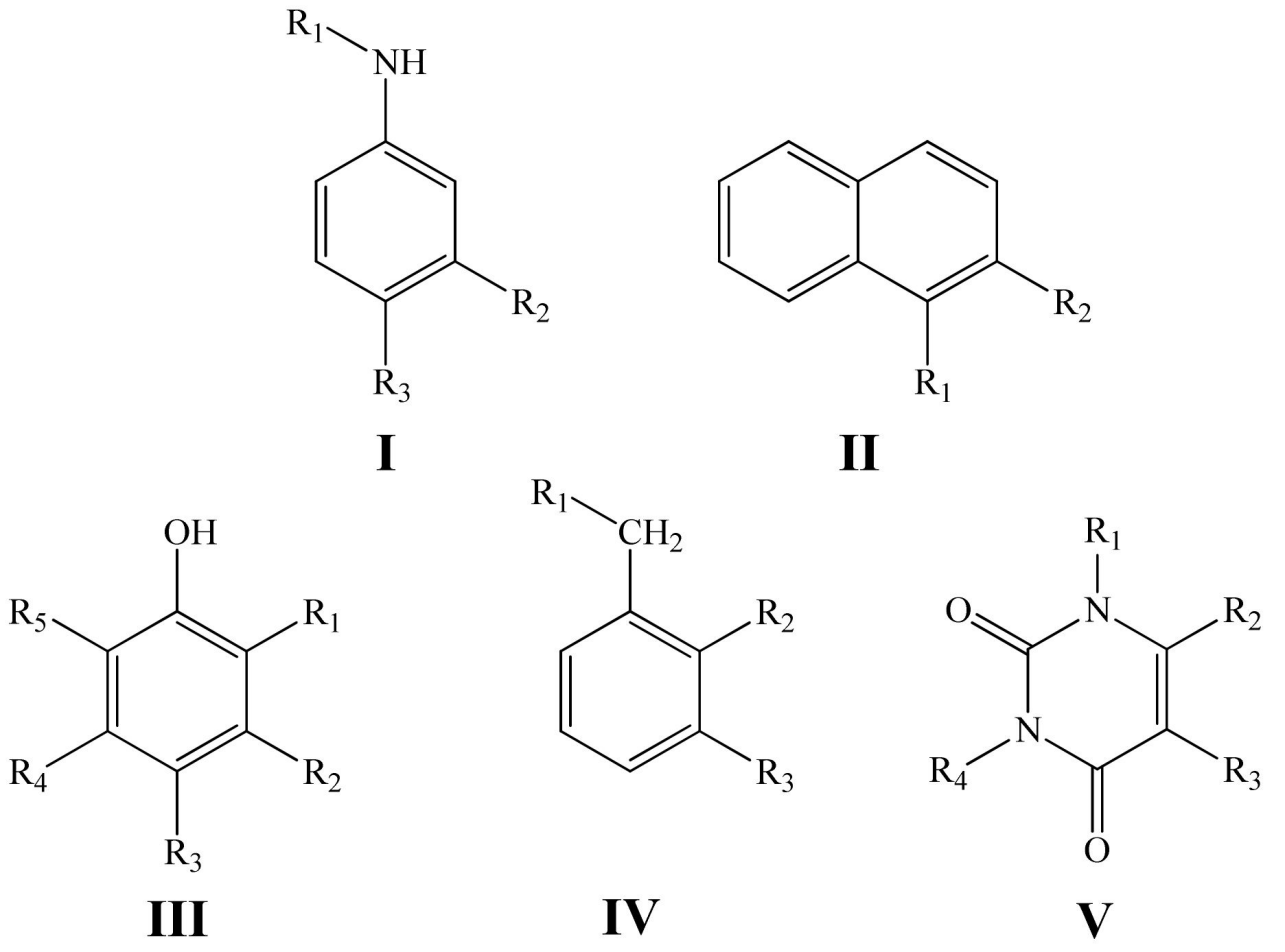
General structural formulas of the inhibitors under study.

In the present work, we have studied a quantitative structure—antioxidant activity relationship in the series of phenols, aminophenols, aromatic amines and uracils with general structural formulas **I**–**V** (Figure 1) using the GUSAR 2013 program and constructed the corresponding statistically significant QSAR models for predicting the k_7_ values.

## 2. Computational Details

### 2.1. Computational Methodology

The structures of the phenol, aminophenol and uracil derivatives selected for the QSAR modeling in GUSAR 2013 [35,36,37,42,43,44,45,46,47] are shown in Figure 1. A complete list of compounds with their experimental k_7_ values is presented in Supplementary Materials (Table S2). The experimental data k_7_ of phenol, aminophenol and uracil derivatives (in L·mol^−1^·s^−1^) were selected from the literature [27,48,49] and converted to logarithmic values (lgk_7_) for QSAR analysis.

**Figure 2:**
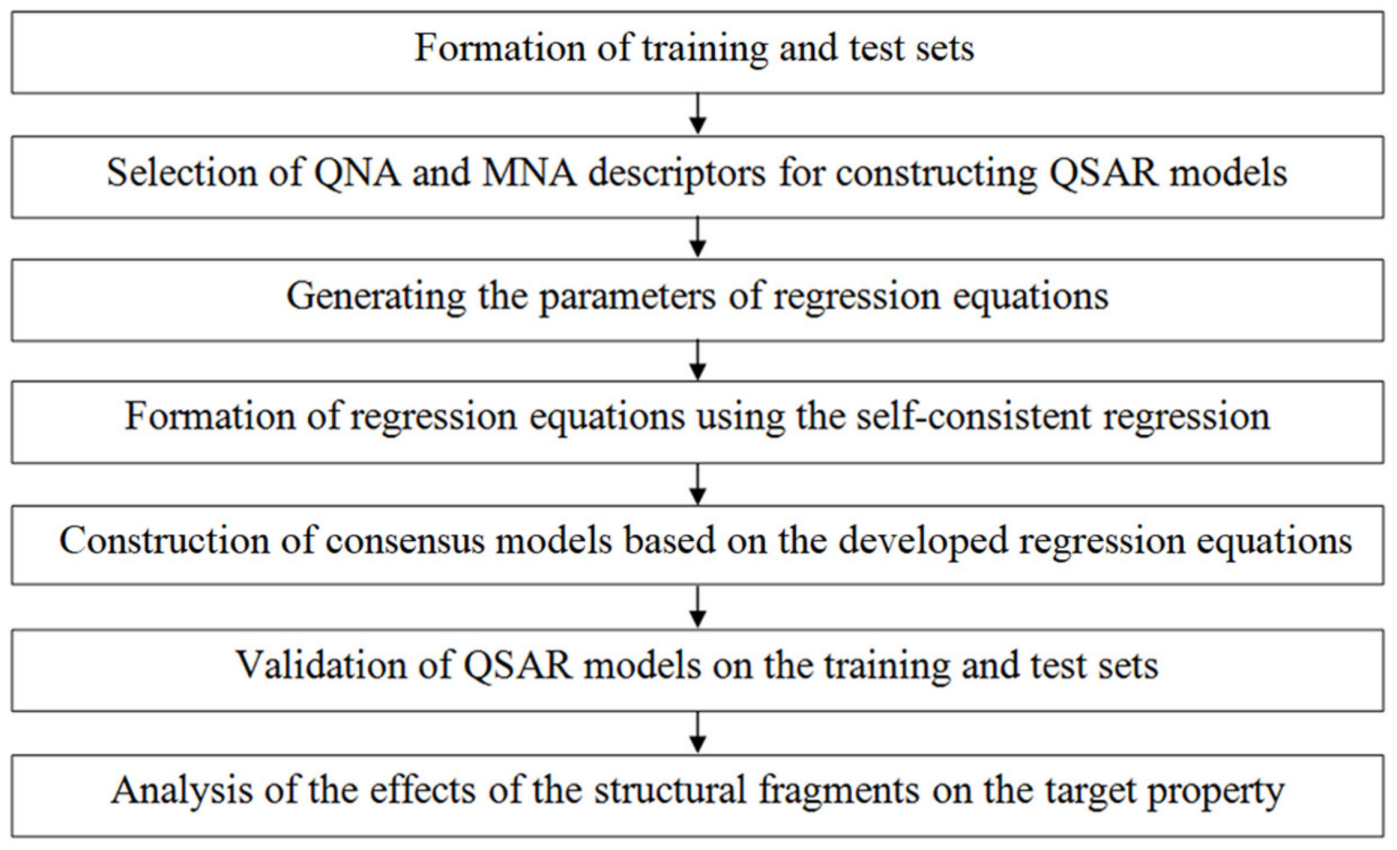
Schematic representation of the GUSAR algorithm.

The QSAR models were built in several steps schematically presented in Figure 2.

#### Formation of Training and Test Sets

**Figure 3:**
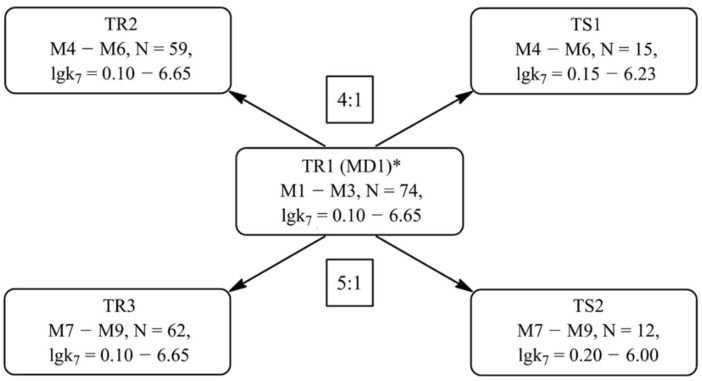
The scheme for the formation of training and test sets for the further development of QSAR models (MD is an array of the data, TR is a training set, TS is a test set, N is the number of compounds included in the corresponding set). * MD1 is formed on the data taken from [48,49,50].

Training (TR1–TR3) and test (TS1–TS2) sets for the development of the models M1–M9 were composed based on the set of 74 compounds (MD1) according to the scheme shown in Figure 3. Set MD1 is made up with the uracil, phenol and aminophenol derivatives, for which the trustworthy k_7_ values are known [48,49,50]. Thus, the k_7_ values are used as a quantitative parameter for assessing target AOA.

The M1–M3 models were built based on the set TR1, which contains a complete set of antioxidant structures of the MD1 set and their corresponding lgk_7_. The TR2 training set is designed to develop QSAR models M4–M6 and includes 59 antioxidant structures. The TS1 set was used to test the predictabilities of models M4–M6. Both of these sets were obtained by the separation of the TR1 set in the ratio 4:1, i.e., each fifth compound was transferred to TS1 from TR1. Before this, all the structures of TR1 were ranked according to the increase in lgk7 parameter. The features of sets TR1–TR2 and TS1 are presented in Table 1 and Table 2. The statistical characteristics presented in these tables indicate a fairly uniform distribution of data in all training and test sets. The average values of the lgk7 parameter and the range of its variation in all training and test sets are numerically close. The observed response values lgk7 of training sets TRi and test sets TSi are similarly distributed around training mean. These facts indicate the correctness of the formation of training and test sets.

The TR3 set contains 62 inhibitors for the further development of QSAR models M7–M9. The validity of these models was assessed using test set TS2. Both of these sets were formed as above, viz. based on TR1. However, in this case, the TR1 set was divided in ratio 5:1, so that each sixth compound of TR1 is transferred to TS2. The characteristics of sets TR3 and TS2 are presented in Table 1 and Table 2.

The structures of the TR1–TR3 and TS1–TS2 sets were generated in the Marvin Sketch 17.22.0 program [51] and converted to the SDF format using the Discovery Studio Visualiser [52].

The variation of the lgk_7_ values within the training sets are Δlgk_7_ > 6. Thus, the condition for development of reliable QSAR models is fulfilled [53].

### 2.2. QSAR Model Development

The QSAR models M1–M9 for quantitative prediction of the antioxidant activity of uracil, phenol, and aminophenol derivatives were based on two types of substructural descriptors of atomic neighborhoods: QNA (Quantitative Neighborhood of Atoms) and MNA (Multilevel Neighborhoods of Atoms) [35,36,37,42,43,44,45,46,47]. These descriptors are automatically computed in the GUSAR2013 program based on the structural formulas of chemical compounds, taking into account the valence and partial atomic charges. The peculiarities of chemical bonds are not considered. Let’s briefly explain the ideology of calculating descriptors in the program GUSAR2013. The ideology of calculating QNA and MNA descriptors is described in detail Supplementary Materials and papers [35,36,37,38,39,40,41,42,43,44,45,46]. Additionally, three descriptors of the whole molecule (topological length, topological volume, and lipophilicity) were used to enhance the descriptive and predictive ability of the models.

In the GUSAR 2013 program, the description of the structure and the calculation of the regression coefficients for the further construction of QSAR models is based on the use of two types of substructural descriptors of atomic neighborhoods: MNA (Multilevel Neighborhoods of Atoms) and QNA (Quantitative Neighborhoods of Atoms) [39,40]. They are automatically deduced from the matrices of molecular connectivity, standard ionization potentials (IP) and electron affinities (EA). The QNA descriptors are defined by two functions, P and Q. The P and Q values for each atom i are calculated using the following formulae [39]:

(1) $$P_{i}=B_{i}\sum_{k}{\left(\exp\left(-\frac{1}{2}C\right)\right)}_{\mathrm{ik}}B_{k}$$

(2) $$Q_{i}=B_{i}\sum_{k}{\left(\exp\left(-\frac{1}{2}C\right)\right)}_{ik}B_{k}A_{k}$$

(3) $$A_{k}=\frac{1}{2}\left({\mathrm{IP}}_{k}+{\mathrm{EA}}_{k}\right){,}_{}B_{k}={\left({\mathrm{IP}}_{k}-{\mathrm{EA}}_{k}\right)}^{-1/2}$$

where k is the remaining atoms in the molecule, IP is the first ionization potential, EA is the electron affinity for each atom (in eV), and C is the connectivity matrix for the molecule as a whole [46]. The standard values IP and EA of atoms in a molecule were collected from the literature. A detailed description of QNA descriptors is represented in [45].

Thus, the QNA descriptors are calculated taking into account the relationships between all atoms of the structure. These values describe each atom of the molecule but, at the same time, depend on the structure of the molecule as a whole [45,46]. The QNA values are the basic information for calculating the Chebyshev 2D polynomials. It is important to note that in the final QSAR models, the independent variables include the mean values of the individual two-dimensional Chebyshev polynomials from the P and Q values calculated for all atoms in the molecule. Thus, the regression equations constructed in the GUSAR 2013 program take into account both the specificity and physicochemical properties of each atom entering the training set [41,43,44,45,46]. However, QNA descriptors cannot be physically interpreted due to the peculiarities of their calculation. In this regard, they are not explicitly displayed under calculations.

The MNA descriptors are computed using the PASS algorithm (Prediction of Activity Spectra for Substances) [39,40], which predicts approximately 6400 “biological activities” with an accuracy threshold of an average prediction of at least 95%. These descriptors are generated based on the structural formulae of chemical compounds without using any pre-compiled list of structural fragments [39,40,41,46]. The authors of the GUSAR 2013 program report that “MNA-descriptors are based on the molecular structure representation, which includes hydrogens according to the valences and partial charges of other atoms and does not specify the types of bonds.” They are generated as “a recursively defined sequence:

- zero-level MNA descriptor for each atom is the mark A of the atom itself;
- any next-level MNA descriptor for the atom is the substructure notation A (D_1_D_2_…D_i_…), where Di is the previous-level MNA descriptor for i–th immediate neighbor of the atom A.

The neighbor descriptors D_1_D_2_…D_i_… are arranged in a unique manner. This may be, for example, a lexicographic sequence. MNA descriptors are generated using an iterative procedure, which results in the formation of structural descriptors that include the first, second, etc. neighborhoods of each atom. The label contains not only information about the type of atom, but also additional information about its belonging to a cyclic or acyclic system, etc. For example, an atom that does not enter a ring is marked with a “―“.

Based on the MNA descriptors using B-statistics, calculated in the PASS program, the biological activity spectrum of a chemical compound is predicted [35,36,42,43,44].

The output of the PASS program is the probabilities of the activity (Pa) and of inactivity (Pi) of each prognostic result. The difference between these two values (Pa–Pi) for a randomly selected subset of predicted activities is used as independent variables for regression analysis in GUSAR. GUSAR2013 incorporates a PASS version that pedicts 4130 types of biological activity. The developers of the GUSAR 2013 program report that the list of predictable biological activities currently includes 501 pharmacotherapeutic effects, 3295 mechanisms of action, 57 adverse and toxic effects, 199 metabolic terms, 49 transporter proteins and 29 activities related to gene expression [46]. The average accuracy of a reliable prediction of biological activity, calculated by leave-one-out cross-validation procedure is approximately 95% [54]. However, the regression equation constructed based on the MNA descriptors reveals the specificity of the action of the compound but does not explicitly reflects the physicochemical parameters of chemical compounds [46].

In addition, the GUSAR 2013 program calculated the QSAR descriptors of an entire molecule such as topological length, topological volume, lipophilicity, and physicochemical descriptors (numbers of positive and negative charges, number of donors and acceptors of the hydrogen bond, number of aromatic atoms, molecular weight and number of halogen atoms) [39,40]. These parameters were added to the QNA descriptors. The topological length of a molecule was calculated as the maximal distance between any two atoms and the volume of a molecule as the sum of each atom’s volume, 4/3 π R^3^, where R is the atomic radius [45].

The authors of the GUSAR 2013 program report that “in GUSAR, the scale of QNA- and PASS-based descriptors ranges from −1 to 1. Therefore, no additional normalization is required for these types of descriptors. Only whole-molecule descriptors are normalized using a standard Z-score normalization procedure” [40].

It should be noted that the program is able to construct QSAR models both relying solely on one of these types of descriptors, and on their combination in terms of the consensus approach [42,43,44]. At the same time, based on the consensus approach methodology, models for quantitative prediction of biological activity for these descriptors are calculated independently of each other. The examples of the sample QSAR GUSAR models for predicting the toxic effects of chemical compounds are available free via the link http://www.way2drug.com/GUSAR.

However, it noteworthy that the features of the QNA and MNA calculations retain these descriptors without unambiguous physical interpretation. For this reason, in the commercial and academic versions of the GUSAR 2013 program for broad use, the regression equations are not displayed.

To reduce the descriptor space and select the most significant descriptors, the RBF-SCR method was used, which combines the advantages of the radial basis function (RBF) interpolation and the self-consistent regression (SCR) method. It has a 3-step algorithm:

(1) Selecting descriptors using the SCR method. This is a regularized method of the least squares. Independent parameters *a* are calculated in this method according to the Equation (4) [43]:
  (4) $$a=\mathrm{ArgMin}\left[\overset{n}{\underset{i=1}{\sum }}y_{i}-\overset{m}{\underset{k=0}{\sum }}x_{ik}a_{k}{)}^{2}+\overset{m}{\underset{k=1}{\sum }}v_{k}a_{k}^{2}\right]$$
where a is the regression coefficient, n is the number of objects, y_i_ is the response value of the i-th object, m is the number of independent variables, x_ik_ is the value of the k-th independent variable of the i-th object, a_k_ is the k-th value of the regression coefficients, and v_k_ is the k-th value of the regularization parameters. Equation (4) has the following solution:
  $$a={\mathrm{TX}}^{T}y{,}_{}\;T={\left(X^{T}X+V\right)}^{-1}$$
where X^T^ is the transposed regression matrix X, and V is the diagonal matrix of the regularization parameters. The regression coefficients obtained from the SCR reflect the contribution of each particular descriptor (variable) to the final equation. The higher the absolute value of the coefficient, the greater its contribution. Thus, the regression coefficients obtained after the SCR can be used to weight the descriptors (variables) depending on their importance.
(2) Calculating the radial basis functions using the weighted coefficient of SCR as a criterion of similarity. The RBF-SCR method can be expressed as [39]:
  (5) $$y\left(x\right)=\overset{N}{\underset{i=1}{\sum }}w_{i}\varphi \left(\left\|ax-a_{i}x_{i}\right\|\right)=\Phi w$$
where a is taken from Equation (4).
  The weights w are calculated as:
  (6) $$w=\Phi^{-1}y$$
(3) Calculating the weighting coefficients RBF by the least squares.

The authors of the GUSAR 2013 program report [39] that, in contrast to the RBF network, in the RBF-SCR method each input variable is used as a center of gravity. The learning process is performed on all input variables of the training set. The RBF-SCR interpolation is based on a linear radial basis function that allows modeling a variety of training sets with a high level of dissimilarity between the objects.

It is shown that such a combined method of selecting descriptors in regression models provides more accurate prediction results as compared with the individual SCR and RBF methods, even if they are used in the consensus approach [39]. A detailed description of this method is available in Supplementary Materials and paper [39].

Additionally, the adequacy of the constructed models was tested using cross-validation procedure with 20-fold randomized release of 20% of compounds from the training sets (20-fold CV, leave-many-out procedure). Both procedures are automatic in the GUSAR 2013 program [35,36,37,38,39,40,41,42,43,44,45,46,47].

Each of the six final QSAR consensus models M1–M2, M4–M5, M7–M8 include 20 partial regression equations automatically united based on their similarity. M1, M4, M7 models were built based on the QNA descriptors with automatic addition of the topological lengths, topological volumes and lipophilicities of the simulated antioxidant structures. M2, M5, M8 models were similarly built based on the MNA descriptors with addition of the abovementioned molecular descriptors. Models M3, M6 and M9 were built guiding the same principle. Each of these models included 320 partial regressions, which were built independently of each other based on QNA or MNA descriptors. Note that the regression equations operating with QNA and MNA descriptors have no direct physical interpretation and, therefore, are not displayed in GUSAR 2013. The final prediction of the lgk_7_ values for a particular compound is formed on the averaged predicted lgk7 values of the partial QSAR regression models.

Applicability domain estimation of the constructed models was performed with three different approaches: similarity, leverage and accuracy described in detail in Supplementary Materials.

As we have built the QSAR consensus model containing 20–100 single models, it is impossible to provide a general equation that would allow describing all selected variables. For this reason, such QSAR models cannot provide information about positive and negatively affecting descriptors. Instead, the GUSAR program allows evaluating the contribution of each atom of the structure to the target property.

### 2.3. Assessment of the Descriptive and Predictive Ability of QSAR Models

The descriptive ability of the QSAR models M1–M9 and the corresponding systematic errors were estimated by the predicted lgk_7_ values for the structures of TR1–TR3 using a metrics based on the determination coefficients of R^2^ (R^2^_TSi_, R^2^_0_, $\bar{R_{\mathrm{mTSi}}^{2}}$) and based on the concordance correlation coefficient (CCC). The prognostic ability of these models was assessed with the predicted lgk_7_ values for the structures of test sets TS1 and TS2 using the metrics of two categories: (1) based on the determination coefficients R^2^ (R^2^_TSi_, R^2^_0_, Q^2^(_F1_), Q^2^(_F2_), $\bar{R_{\mathrm{mTSi}}^{2}}$ and CCC); or (2) based on the prediction lgk_7_ errors (root mean square error (RMSEP), mean absolute error (MAE) and standard deviation (S.D.)) [55,56,57,58,59]. The calculations of these statistical parameters were performed using the Xternal Validation Plus 1.2 program [60].

Additionally, the prognostic abilities of the QSAR consensus model were estimated by comparing the predicted and known experimental lgk_7_ values for *N*-2-ethylhexyl-*N*′-phenyl-*p*-phenylenediamine (8-PPDA), a promising industrial antioxidant, which was not included in the data array MD1. The experimental lgk_7_ values for this compound were measured by a manometric method based on the absorption of atmospheric oxygen using the model liquid-phase ethylbenzene oxidation initiated by azodiisobutyronitrile (AIBN) at 348 K. The kinetic curves were recorded using a universal manometric differential device [61,62,63,64,65,66].

**Scheme 1:**
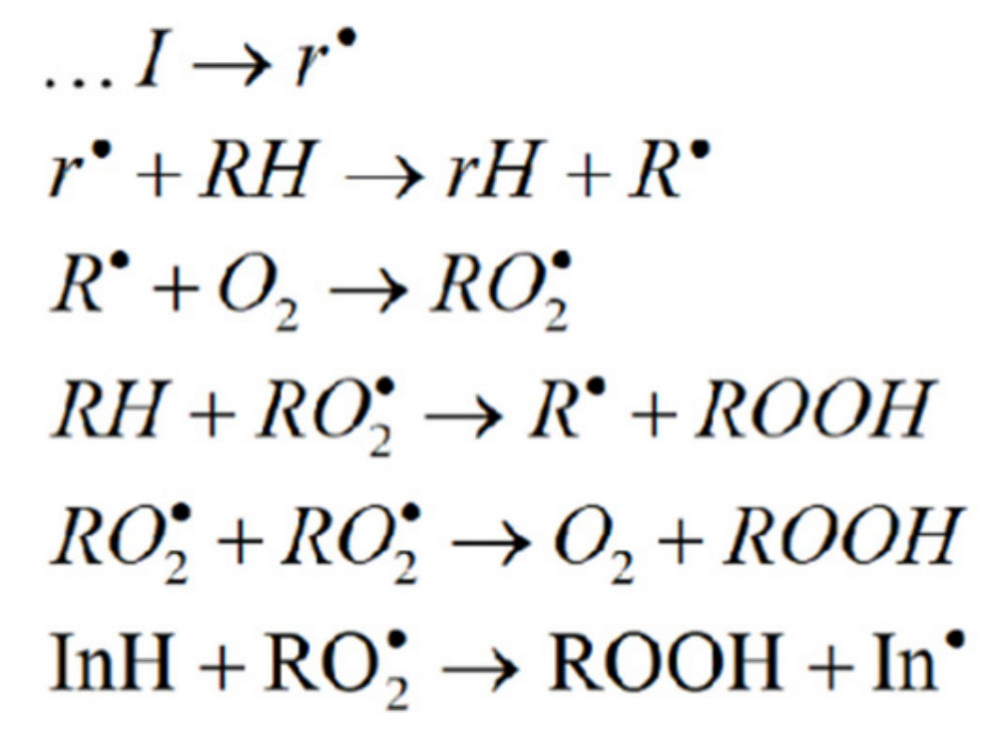
I, RH and InH are the initiator, oxidized substrate and inhibitor, respectively.

In these experiments, the rate constant of chain termination *f*k_7_ was a quantitative parameter of AOA, where *f* is the inhibitor capacity, equal to the number of radical intermediates decaying in the interaction with one inhibitor molecule (8-PPDA) [14]. This kinetic parameter was determined by the concentration effect of 8-PPDA on the oxidation rate of ethylbenzene, a model substrate. When analyzing the experimental data, we used the basic mechanism of the inhibited radical-chain oxidation of organic compounds (Scheme 1) [14,27].

## 3. Results and Discussion

Based on the consensus approach implemented in the GUSAR 2013 program, the quantitative relationship between the structure and antioxidant activity of uracil, phenol and aminophenol derivatives of sets TR1–TR3 was modeled. Depending on the type of the used descriptors, three QSAR consensus models have been built for each of these training sets. The descriptive and predictive abilities of these models were estimated on the structures of TR1–TR3 by cross-validation with 20-fold randomized exception of 20% of compounds and using the structures of the test sets. The descriptive parameters of consensus models M1–M9, calculated automatically in the GUSAR 2013 program based on a comparison of the experimental and predicted lgk_7_ values for these nine models are presented in Table 3. The experimental and predicted lgk_7_ values used for calculating the statistical parameters of models M1–M9 are collected in Supplementary Materials (Tables S3–S7).

Note that Table 3 contains the averaged values of determination coefficients, RMSD and Fisher criterion obtained with all partial regression models included in the consensus model M_i_.

**Figure 4:**
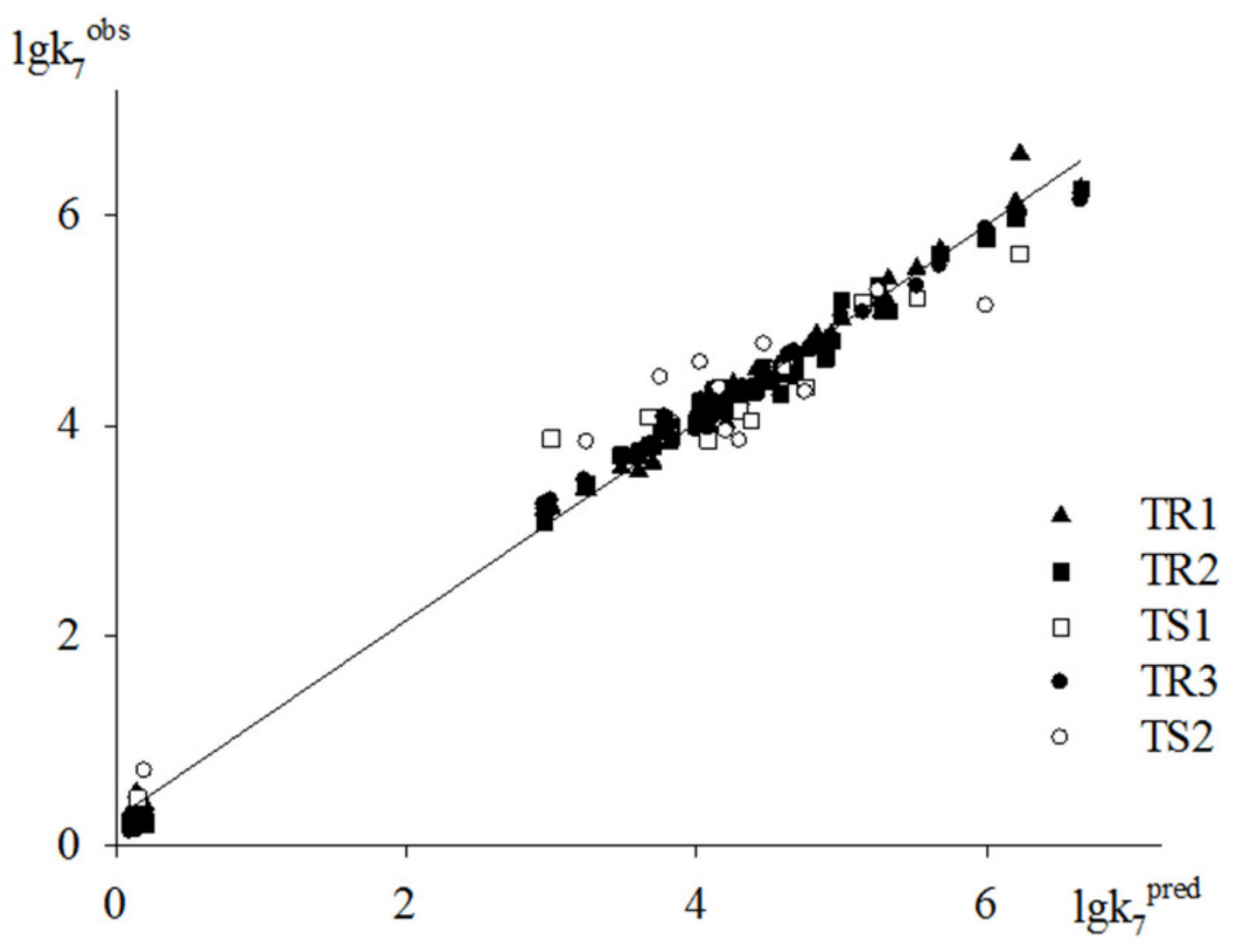
Plot of predicted activities vs. experimental ones based on Models M3, M6 and M9.

To estimate the predictive power of the method underlying the GUSAR 2013 models, we used two test sets of antioxidants. The models obtained via the computation procedures with three training sets are used to predict the activity of compounds from TS1 and TS2. As an example, the plot of the predicted lgk_7_ values based on models M3, M6 and M9 versus experimental ones is shown in Figure 4. Dependencies depicted in this figure clearly indicate a fairly high descriptive and predictive ability of models M3, M6 and M9.

Meanwhile, the averaged values of the coefficients of determination, standard deviation and the Fisher criterion are not sufficient for the reliable characterization of the descriptive and predictive abilities of models M1–M9. Therefore, according to recommendations [39,58,59], we used the metrics of two categories in addition to the GUSAR 2013 parameters. These are (1) metrics based on the coefficients of determination R^2^ (R^2^_TSi_, R^2^_0_, Q^2^(_F1_), Q^2^(_F2_), $\bar{R_{\mathrm{mTSi}}^{2}}$, CCC); and (2) metrics that allow estimating prediction errors of lgk_7_ values (RMSEP, MAE, S.D.) [53,55,56,57,58,59]. These statistical parameters were calculated using the Xternal Validation Plus 1.2 program [60]. Additionally, in this program, we estimated the systematic errors of the models.

We considered that the QSAR model M_i_ possesses a high descriptive ability if its determination coefficients of different types for 95% of the data TR_i_ are close to each other and tend to unity.

We considered that the QSAR model M_i_ possesses a high predictive ability if the following four conditions are simultaneously fulfilled for 95% of the test sets:

(1) determination coefficients R^2^, R^2^_0_, R^2^′_0_, Q^2^_F1_, Q^2^_F2_ and CCC criterion are close to each other and tend to unity;
(2) R^2^_m_ > 0.5 if ΔR^2^_m_ < 0.2;
(3) MAE value does not exceed 10% of the Δlgk_7_ interval of the compounds from TR_i_;
(4) the sum MAE + 3·S.D. does not exceed 20% of the Δlgk_7_ interval of the compounds from TR_i_.

We considered that the QSAR model M_i_ has a low predictive ability if the following four conditions are simultaneously fulfilled for 95% of the test sets:

(1) determination coefficients R^2^, R^2^_0_, R^2^′_0_, Q^2^_F1_, Q^2^_F2_ and CCC criterion do not exceed the threshold value equal to 0.6;
(2) R^2^_m_ ≤ 0.5 if ΔR^2^_m_ ≤ 0.2;
(3) MAE value is higher than 15% of the Δlgk_7_ interval of the compounds from TR_i_;
(4) the sum MAE + 3·S.D. is larger than 25% of the Δlgk_7_ interval of the compounds from TR_i_.

Otherwise, we considered the descriptive and predictive abilities of the models as moderate.

**Figure 5:**
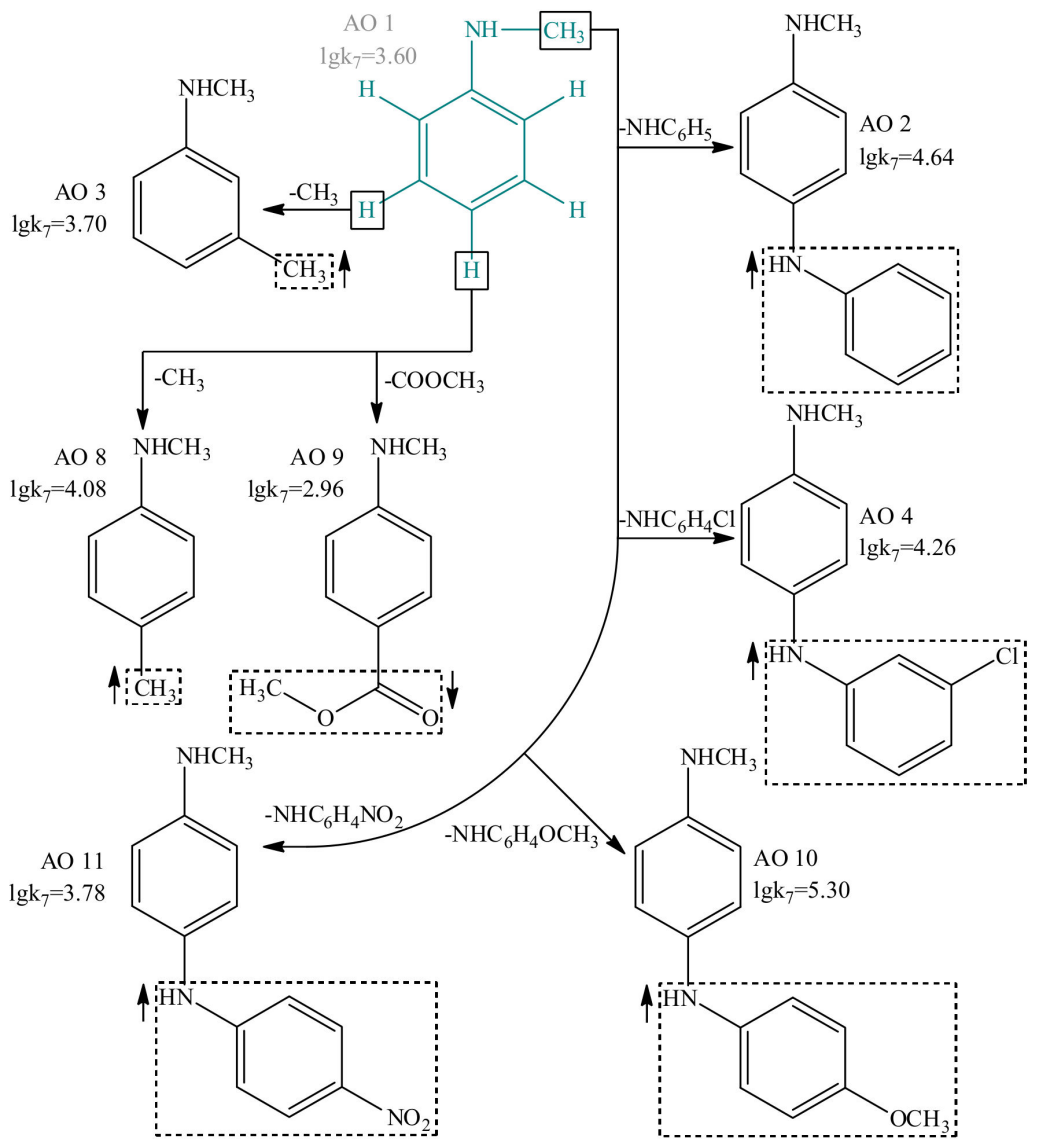
The effect of structural features on the antioxidant activity of compounds with general structural formula I.

**Figure 6:**
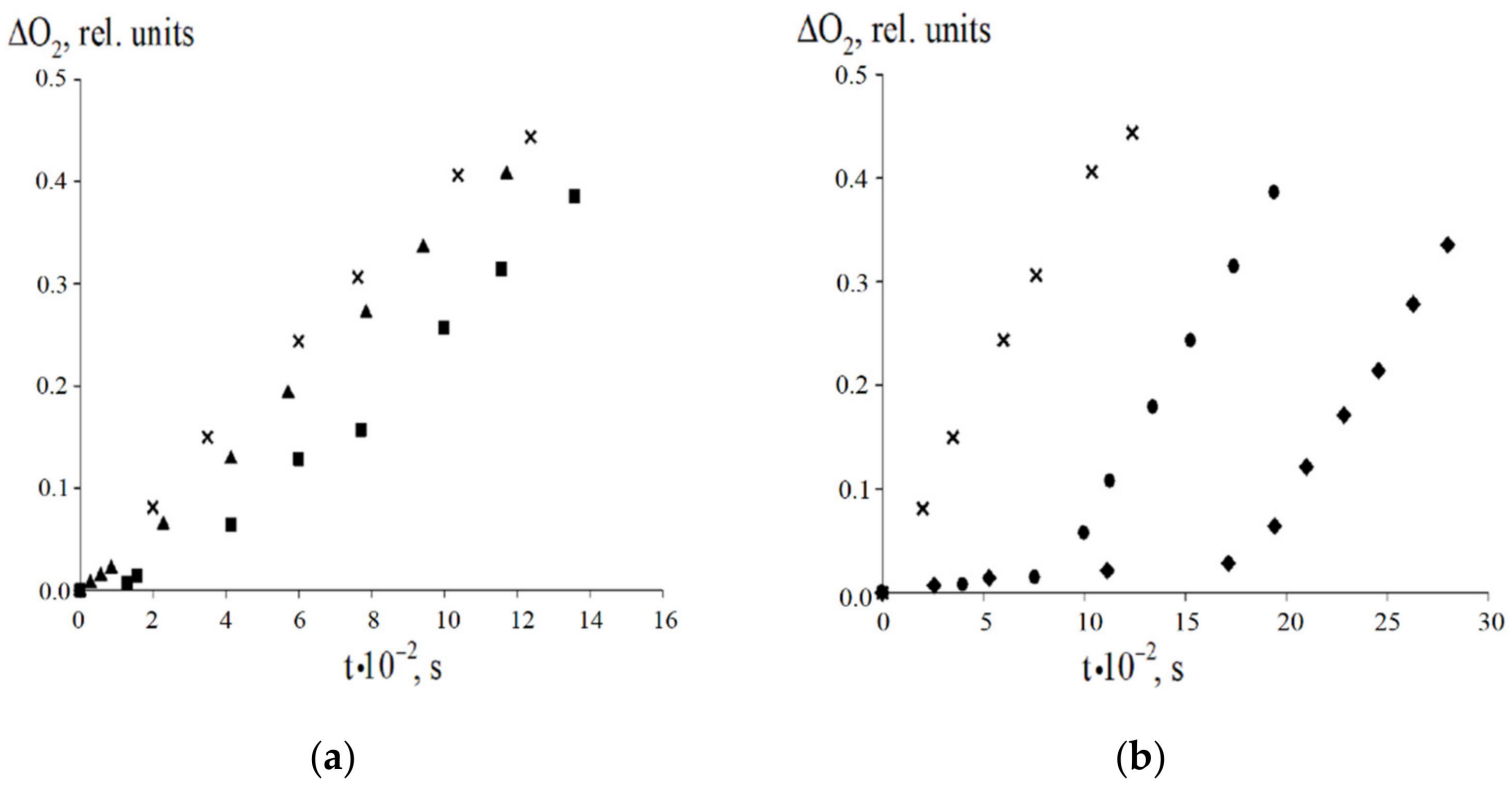
The kinetic curves of the oxygen absorption upon the ethylbenzene oxidation (V_i_ = 2.4·10^−7^ mol·L^−1^·s^−1^, 348 K) without (**×**) and with 8-PPDA: (**a**) 2 × 10^−5^ mol·L^−1^ (▲) and 4.03 × 10^−5^ mol·L^−1^ (■); (**b**) 1 × 10^−4^ mol·L^−1^ (●) and 1.61 × 10^−4^ mol L^−1^ (♦).

Statistical criteria of the descriptive and predictive abilities of the QSAR models M1–M9 deduced from comparing experimental and predicted lgk_7_ are presented as diagrams (Figure 5 and Figure 6). The full set of the calculated statistical parameters for the TR1–TR3 and TS1–TS2 structures is available as Supplementary Materials (Tables S3–S7). The diagrams demonstrate that models M1–M9 possess high descriptive and predictive abilities.

A slight difference between the different determination coefficients in combination with appropriate MAE and MAE + 3·S.D. values (Table 4 and Table 5) suggests that valid QSAR models for the AOA prediction can be built using one specific type of descriptors (QNA/MNA) or their combinations.

In addition, using the GUSAR 2013 program and comparative analysis of the experimental data, we have systematically studied model antioxidants I–V and elucidated the functional groups, which are able to modulate the lgk_7_ values. Figure 5 shows the structures of 7 compounds with the general structural formula I used for the comparative analysis. More detailed information on the effect of functional groups on the antioxidant activity of compounds with the general structural formulas I-V is presented in Supplementary Materials (Figures S1–S10, Tables S8–S9).

In general, the results of the structural analysis using the GUSAR 2013 program are consistent with the literature data on the effect of various ortho, meta, and para substituents on the antioxidant activity of compounds I–V. Particularly, the introduction of electron-donor substituents in the ortho, meta, and para positions of I–IV increases the lgk_7_ parameter. At the same time, electron-acceptor substituents in the same positions lead to the opposite effect. In compounds V, the effect of electron-donor substituents is ambiguous. Methyl groups in positions R_1_, R_2_, R_3_ increase the nominal value of lgk_7_ but replacing the hydrogen atom from positions R_1_ and R_2_ by the hydroxyl or amino group, in contrast, reduces the antioxidant properties. A detailed description of this fact is presented in Supplementary Materials (Figure S6). Uracil derivatives with electron-acceptor substituents in R_1_, R_2_, R_3_ and R_4_ positions are not used in the modeling, and for this reason not discussed [61].

Then we have applied the consensus models M3, M6 and M9 to predicting the lgk_7_ parameter of 8-PPDA, an aromatic amine antioxidant. We choose these models for this purpose as they are based on the descriptors of different types and, therefore, expected to provide more accurate estimates. The results of the lgk7 calculation for 8-PPDA within the M3, M6 and M9 models are shown in Table 6.

Note that compound 8-PPDA has already used as an antioxidant in composite synthetic rubbers [67]. However, its antioxidant activity using classical model reactions of liquid-phase oxidation of ethylbenzene or cumene has not been studied. Thus, we complement our calculations with the corresponding experiments to measure rate constant k_7_.

We have studied the antioxidant properties of 8-PPDA under the initiated radical-chain oxidation of ethylbenzene in the kinetic mode at 348 K (azodiisobutyronitrile is the initiator of the oxidative process). The kinetic curves demonstrate that the introduction of 8-PPDA into the reaction system decreases the rate of the oxygen absorption measured in the induction period (Figure 6, Table 7).

**Figure 7:**
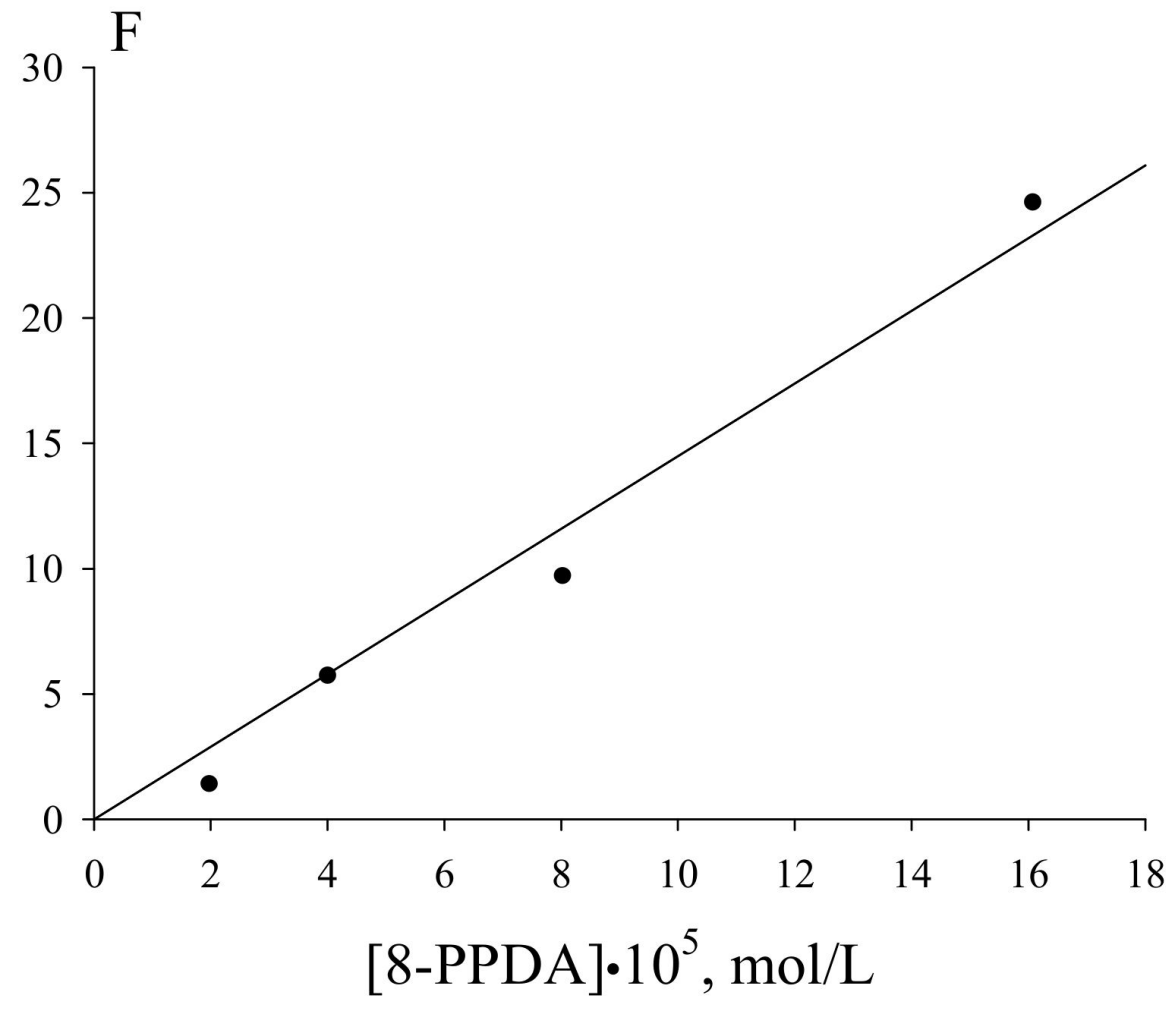
Dependence of the inhibition parameter on the 8-PPDA concentrations (V_i_ =2.4·10^−7^ mol·L^−1^·s^−1^, 348 K); the correlation coefficient R = 0.95.

As follows from Figure 7, the inhibition parameter F deduced from the initial rates of the inhibited oxidation linearly depends on the 8-PPDA concentration in the range of its concentrations (0.2–1.61)·10^−4^ mol·L^−1^:

(7) $$F=\frac{V_{0}}{V}-\frac{V}{V_{0}}=\frac{{\mathrm{fk}}_{7}\left[\mathrm{InH}\right]}{\sqrt{2k_{6}V_{i}}}$$

where V and V_0_ are the rates of the oxygen absorption with and without 8-PPDA in the reaction system, respectively.

**Figure 8:**
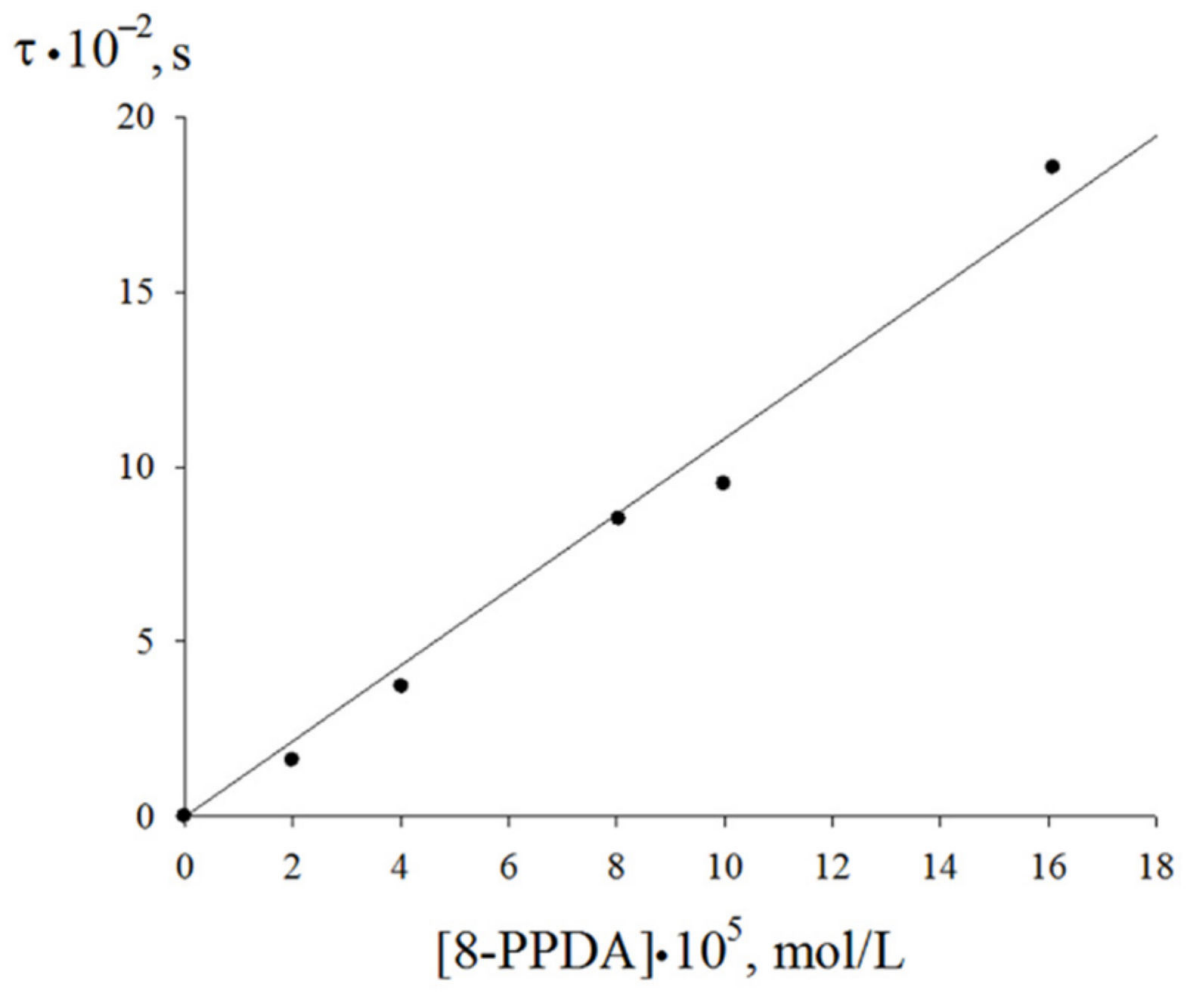
Dependence of the induction period on the initial concentration of the inhibitor (348 K, V_i_ = 2.4·10^−7^ mol·L^−1^·s^−1^).

The linearization in the coordinates of Equation (7) lead to the effective rate constant of the chain termination by the 8-PPDA molecule fk^exp^_7_ = (4.8 ± 0.2)·10^5^ mol·L^−1^·s^−1^. The induction period (τ) on the oxygen absorption curves corresponding the 8-PPDA-inhibited ethylbenzene oxidation (Figure 6) linearly depends on the inhibitor concentration. This dependence (Figure 8) allows assessing the stoichiometric inhibition coefficient via Equation (8):

(8) $$\tau =\frac{f\left[\mathrm{InH}\right]}{V_{i}}$$

where τ is the induction period on the kinetic curves of oxygen absorption upon the oxidation of ethylbenzene inhibited by 8-PPDA and V_i_ is the initiation rate of the oxidation process. The calculation according to Equation (8) gives f = 2. Thus, the inhibition rate constant k_7_^exp^ for 8-PPDA can be calculated as:

(9) $$k_{7}^{\exp}={\mathrm{fk}}_{7}^{\exp}/f$$

where f is the antioxidant capacity that equals the number of radical intermediates cease to exist in the interaction with one antioxidant molecule.

A comparative analysis of the predicted values (lgk_7_^pred^) with the experimental one (lgk_7_^obs^) demonstrates a high predictability of the QSAR consensus models M3, M6 and M9. Hence, they can be applied to screening novel antioxidant structures.

Thus, all QSAR consensus models M1–M9 have descriptive and predictive ability as follows from the comparison of experimental and predicted lgk_7_ values for the TR1–TR3 structures, external and internal TS1 and TS2 test sets, and 8-PPDA. These models can be used for screening on virtual libraries and databases to find new antioxidants among substituted phenols, polyphenols, aminophenols, aromatic amines and uracils.

Thus, the approach implemented in the GUSAR 2013 program have been previously used only for modeling the biological activity of low-molecular compounds. We have shown that it allows simulating the key kinetic parameter of antioxidant activity (k_7_) with high accuracy and this program could be recommended as an auxiliary tool when searching for new antioxidants.

## 4. Conclusions

Based on the QSAR methodology implemented in the GUSAR 2013 program, we have studied a quantitative structure–antioxidant activity relationship in the set of 74 organic compounds (phenol, aminophenols, aromatic amines and uracil derivatives; see structural formulas I–V), which have a different degree of antioxidant activity expressed through the lgk_7_ parameter (lgk_7_ 0.01 ÷ 6.65 for these compounds). Operating with MNA/QNA descriptors, descriptors corresponding to the whole molecule (topological length, topological volume and lipophilicity), RBF-SCR method, we have developed 9 statistically significant QSAR consensus models. The QSAR models demonstrate high accuracy in predicting the lgk_7_ values for the compounds of training sets and appropriately predict lgk_7_ for the test sets (R^2^_TR_ > 0.6; Q^2^_TR_ > 0.5; R^2^_TS_ > 0.5). We recommend using the QSAR models M3, M6 and M9 for virtual screening of new antioxidants because they are based on a combination of the descriptors different types. This guarantees reliability of the predicted lgk_7_ values.

However, it should be borne in mind that the QSAR consensus models we have developed are not universal. These models are able to adequately predict the numerical values of the lgk_7_ parameter only for antioxidants with general structural formulas I–V. At the same time, the range of correct prediction of the lgk_7_ parameter using all the QSAR models developed by us, including the M3, M6, M9 models, is quite wide. The experimental values of the lgk_7_ parameter for the modeled antioxidants, on the basis of which all QSAR models were developed and their validity was assessed, varied from 0.01 to 6.65.

In general, the results of the structural analysis using the GUSAR 2013 program are fully consistent with the literature data on the effect of various ortho, meta, and para substituents on the antioxidant activity of phenols. The introduction of electron-donor substituents into the ortho, meta, and para positions of compounds I–IV increases the lgk_7_ parameter. Electron-acceptor substituents in the same positions reduce the antioxidant activity of phenols. In uracils with the general structural formulas V, the effect of electron-donor substituents is ambiguous. The introduction of methyl groups in the positions R_1_, R_2_, R_4_ increases the nominal value of the parameter lgk_7_ but replacing the hydrogen atom from R_3_ by the hydroxyl or amino group, in contrast, leads to a decrease in the antioxidant properties (see Supplementary Materials, Figures S1–S6).

A good agreement of the predicted and experimental lgk_7_ values for the compounds of the test sets and 8-PPDA, a ”young” member of the inhibitors family, indicates that the GUSAR 2013 algorithms are applicable to simulating the kinetic parameters of the model liquid-phase oxidation reactions of organic hydrocarbons. Thus, the GUSAR 2013 program allows modeling kinetic parameters relating to non-specific activity in addition to the ADMET properties and diverse biological activities mentioned in the introduction. Previously, we have reported the first results of a successful QSPR simulation of nonspecific activity using the GUSAR 2013 program. In the previous study [68], we have demonstrated the efficiency of the GUSAR descriptors for simulating the photovoltaic performances of the methanofullerene derivatives (we built six statistically significant QSPR consensus models for predicting the power conversion efficiencies of the methanofullerene-based organic solar cells). This QSPR study develops the idea of successfully applying the GUSAR approaches to describing nonspecific activities of the compounds. Our studies in this area will be continued.

In summary, the approach used in the GUSAR 2013 program for constructing regression models demonstrates high performance as applied to both small and large data sets including the antioxidants with different structures. As the program implements a two-dimensional approach, there is no need to search through the entire conformational space and select the most bioactive conformation of the modeled compounds. The mentioned issues open the opportunity for the application of GUSAR 2013 to the antioxidant studies.

This work was supported by grant No. 19-73-20073 of the Russian Science Foundation.

## Figures and Tables

**Table 1:** Statistical characteristics of training sets TR1–TR3.

Value	Designation of TR_i_	Code of the Set		
		TR1	TR2	TR3
Number of compounds	N	74	59	62
Mean value of lgk_7_	$\bar{{\mathrm{lgk}}_{7}}$	3.3750		
Spread of lgk_7_	Δlgk_7_	6.5500		
Distribution of the observed response values of training sets TRi around training mean (in %)	$\bar{{\mathrm{lgk}}_{7}}\pm 0.5$ (%)	20.2703	20.0000	16.6667
	$\bar{{\mathrm{lgk}}_{7}}\pm 1.0$ (%)	50.0000	46.6667	50.0000
	$\bar{{\mathrm{lgk}}_{7}}\pm 1.5$ (%)	72.9730	73.3333	75.0000
	$\bar{{\mathrm{lgk}}_{7}}\pm 2.0$ (%)	83.7838	80.0000	83.3333
	0.10 × Δlgk_7_	0.6550		
	0.15 × Δlgk_7_	0.9825		
	0.20 × Δlgk_7_	1.3100		
	0.25 × Δlgk_7_	1.6375		

**Table 2:** Statistical characteristics of test sets TS1–TS2.

Value	Designation of TS_i_	Code of the Set	
		TS1	TS2
Number of compounds	N	15	12
Mean value of lgk_7_	$\bar{{\mathrm{lgk}}_{7}}$	4.1727	4.0821
Spread of lgk_7_	Δlgk_7_	6.0800	5.8000
Distribution of the observed response values of test sets TSi around test mean (in %)	$\bar{{\mathrm{lgk}}_{7}}\pm 0.5$ (%)	53.3333	50.0000
	$\bar{{\mathrm{lgk}}_{7}}\pm 1.0$ (%)	73.3333	75.0000
	$\bar{{\mathrm{lgk}}_{7}}\pm 1.5$ (%)	86.6667	83.3333
	$\bar{{\mathrm{lgk}}_{7}}\pm 2.0$ (%)	86.6667	91.6667
Distribution of the observed response values of test sets TSi around train mean (in %)	$\bar{{\mathrm{lgk}}_{7}}\pm 0.5$ (%)	20.0000	16.6667
	$\bar{{\mathrm{lgk}}_{7}}\pm 1.0$ (%)	46.6667	50.0000
	$\bar{{\mathrm{lgk}}_{7}}\pm 1.5$ (%)	73.3333	75.0000
	$\bar{{\mathrm{lgk}}_{7}}\pm 2.0$ (%)	80.0000	83.3333

**Table 3:** Statistical parameters and accuracy of the predicted lgk_7_ values of the compounds from training sets TR1–TR3 within the M1–M9 consensus models (using RBF-SCR). Δlgk_7 (TR1)_ = Δlgk_7 (TR2)_ = Δlgk_7 (TR3)_ = 6.55. ^1^

Training Set	Model	N	N_PM_	$\bar{R^{2}}$	$\bar{F}$	$\bar{S.D.}$	$\bar{Q^{2}}$	V
**QSAR Models Based on the QNA Descriptors**								
TR1	M1	74	20	0.999	10.457	0.525	0.843	22
TR2	M4	59	20	0.999	7.676	0.587	0.799	18
TR3	M7	62	20	0.999	6.059	0.544	0.829	24
**QSAR Models Based on the MNA Descriptors**								
TR1	M2	74	20	0.999	18.207	0.478	0.867	18
TR2	M5	59	20	0.998	11.554	0.556	0.819	15
TR3	M8	62	20	0.999	9.864	0.567	0.810	17
**QSAR Models Based on Both QNA and MNA Descriptors**								
TR1	M3	74	100	0.999	10.744	0.490	0.872	22
TR2	M6	59	100	0.999	7.768	0.559	0.830	18
TR3	M9	62	100	0.999	7.011	0.535	0.845	21

^1^ N is the number of structures in the training set; N_PM_ is the number of regression equations used for the consensus model; $\bar{R_{}^{2}}$ is the determination coefficient calculated for the compounds of TR_i_; $\bar{Q_{}^{2}}$ is the correlation coefficient calculated for the training set with the by cross-validation with exception of one; $\bar{F}$ is the Fisher criterion; $\bar{S.D._{}}$—standard deviation; V is the number of variables in the final regression equation.

**Table 4:** The validation parameters of the QSAR models estimated using the Xternal Validation Plus 1.2 program based on the experimental and predicted lgk_7_ values of the compounds form test sets TS1 and TS2. Δlgk_7(TR1)_ = Δlgk_7(TR2)_ = Δlgk_7(TR3)_ = 6.55; Δlgk_7(TS1)_ = 6.08; Δlgk_7(TS2)_ = 5.80. ^1^

Comments	Prediction Parameters	QSAR Model Used for Predicting lgk_7_								
		TR1			TR2			TR3		
		M1	M2	M3	M4	M5	M6	M7	M8	M9
Classical Metrics (100% data)	R^2^	0.9894	0.9922	0.9923	0.9878	0.9897	0.9899	0.9888	0.9902	0.9893
	R^2^_0_	0.9887	0.9906	0.9894	0.9869	0.9888	0.9876	0.9876	0.9890	0.9871
	R^2′^_0_	0.9878	0.9899	0.9881	0.9860	0.9881	0.9863	0.9866	0.9882	0.9856
	$\bar{R_{m}^{2}}$	0.9538	0.9568	0.9387	0.9520	0.9550	0.9369	0.9493	0.9514	0.9357
	ΔR^2^_m_	0.0120	0.0101	0.0115	0.0128	0.0109	0.0123	0.0122	0.0108	0.0129
	CCC	0.9947	0.996	0.9952	0.9929	0.9942	0.9934	0.9932	0.9942	0.9930
Classical Metrics (after removing 5% data with high residuals)	R^2^	0.9917	0.9939	0.9936	0.9899	0.9921	0.9918	0.9922	0.9936	0.9928
	R^2^_0_	0.9911	0.9932	0.9918	0.9893	0.9914	0.9900	0.9917	0.9931	0.9913
	R^2′^_0_	0.9571	0.9589	0.9420	0.9553	0.9571	0.9402	0.9628	0.9648	0.9467
	$\bar{R_{m}^{2}}$	0.9624	0.9632	0.9470	0.9606	0.9614	0.9452	0.9670	0.9683	0.9511
	ΔR^2^_m_	0.0094	0.0074	0.0089	0.0104	0.0084	0.0099	0.0080	0.0067	0.0085
	CCC	0.9962	0.9973	0.9965	0.9944	0.9955	0.9947	0.9956	0.9962	0.9954
Mean absolute error and standard deviation for test set (100% data)	RMSE	0.1341	0.1195	0.1277	0.1518	0.1372	0.1454	0.1466	0.1364	0.1486
	MAE	0.1014	0.0894	0.0997	0.1132	0.1012	0.1115	0.1060	0.0976	0.1103
	S.D.	0.0920	0.0834	0.0841	0.1020	0.0934	0.0941	0.1021	0.0960	0.1004
	MAE+3·S.D.	0.3774	0.3396	0.3520	0.4192	0.3814	0.3938	0.4123	0.3856	0.4115
Mean absolute error and standard deviation for test set (after removing 5% data with high residuals)	RMSE	0.1284	0.1192	0.1247	0.1326	0.1234	0.1289	0.1180	0.1095	0.1203
	MAE	0.0982	0.0889	0.099	0.1003	0.0910	0.1003	0.0887	0.0812	0.0936
	S.D.	0.0853	0.0821	0.0795	0.0874	0.0842	0.0816	0.0785	0.0741	0.0762
	MAE+3·S.D.	0.3541	0.3352	0.3375	0.3627	0.3436	0.3453	0.3241	0.3036	0.3222
Distribution of prediction errors (in %)	ωN in range 0.10 × Δlgk_7 (TR)_	0.000 ^a^	0.000 ^a^	0.000 ^a^	0.000 ^b^	0.000 ^b^	0.000 ^b^	0.000 ^c^	0.000 ^c^	0.000 ^c^
	ωN in range 0.15 × Δlgk_7 (TR)_	0.000 ^a^	0.000 ^a^	0.000 ^a^	0.000 ^b^	0.000 ^b^	0.000 ^b^	0.000 ^c^	0.000 ^c^	0.000 ^c^
	ωN in range 0.20 × Δlgk_7 (TR)_	0.000 ^a^	0.000 ^a^	0.000 ^a^	0.000 ^b^	0.000 ^b^	0.000 ^b^	0.000 ^c^	0.000 ^c^	0.000 ^c^
	ωN in range 0.25 × Δlgk_7 (TR)_	0.000 ^a^	0.000 ^a^	0.000 ^a^	0.000 ^b^	0.000 ^b^	0.000 ^b^	0.000 ^c^	0.000 ^c^	0.000 ^c^
Prediction quality	-	Good								
Systematic error presence	-	Absent								

^1^ Where R^2^, R^2^_0_, and R′^2^ are determination coefficients calculated with and without taking into account the origin; $\bar{R_{m}^{2}}$ is the averaged determination coefficient of the regression function, calculated using values of determination coefficients on the ordinate axis (R^2^_m_) and using them on the abscissa (R′^2^_m_) respectively; $\Delta R_{m}^{2}$ is the difference between R^2^_m_ and R′^2^_m_; CCC is the concordance correlation coefficient; MAE is the mean absolute error; S.D. is the standard deviation; ωΝis the percentage of training sets TR1–TR3, for which the prediction error is less than the interval proportional to 0.1, 0.15, 0.20, and 0.25 of Δlgk_7_ of training sets TR1 (a), TR2 (b) and TR4 (c).

**Table 5:** The validation parameters of the QSAR models estimated using the Xternal Validation Plus 1.2 program based on the experimental and predicted lgk_7_ values of the compounds form training sets TrS1–TrS3 ^1^; Δlgk_7(TR1)_ = Δlgk_7(TR2)_ = Δlgk_7(TR3)_ = 6.55. ^1^

Comments	Prediction Parameters	QSAR Model Used for Predicting lgk_7_					
		TS1			TS2		
		M4	M5	M6	M7	M8	M9
Classical Metrics (100% data)	R^2^	0.9469	0.9461	0.9388	0.8639	0.8797	0.8876
	R^2^_0_	0.9360	0.9423	0.9260	0.8638	0.8769	0.8737
	R^2′^_0_	0.9164	0.9312	0.9005	0.8413	0.8454	0.8184
	Q^2^_F1_	0.9531	0.9579	0.9454	0.8931	0.9031	0.9006
	Q^2^_F2_	0.9357	0.9423	0.9251	0.8638	0.8766	0.8734
	$\bar{R_{m}^{2}}$	0.8152	0.8592	0.7941	0.7951	0.7754	0.7188
	ΔR^2^_m_	0.0663	0.0570	0.0771	0.1208	0.1155	0.1285
	CCC	0.9634	0.9686	0.9566	0.9263	0.9306	0.9254
Classical Metrics (after removing 5% data with high residuals)	R^2^	0.9710	0.9715	0.9659	0.8846	0.9106	0.9046
	R^2^_0_	0.9643	0.9700	0.9575	0.8840	0.9100	0.8963
	R^2′^_0_	0.8550	0.9051	0.8334	0.8045	0.8043	0.7186
	Q^2^_F1_	0.9731	0.9784	0.9670	0.8959	0.9197	0.9038
	Q^2^_F2_	0.9610	0.9687	0.9521	0.8778	0.9057	0.8870
	$\bar{R_{m}^{2}}$	0.8739	0.9202	0.8562	0.8342	0.8455	0.7694
	ΔR^2^_m_	0.0364	0.0292	0.0437	0.0607	0.0835	0.1035
	CCC	0.9783	0.9834	0.9729	0.9382	0.9505	0.9374
Mean absolute error and standard deviation for test set (100% data)	RMSE	0.3318	0.3145	0.3581	0.4984	0.4744	0.4805
	MAE	0.2664	0.2417	0.2843	0.4167	0.3748	0.4150
	S.D.	0.2048	0.2083	0.2254	0.2856	0.3038	0.2529
	MAE + 3·S.D.	0.8808	0.8666	0.9605	1.2735	1.2862	1.1737
Mean absolute error and standard deviation for test set (after removing 5% data with high residuals)	RMSE	0.2598	0.2326	0.2878	0.4457	0.3914	0.4285
	MAE	0.2254	0.1981	0.2422	0.3735	0.3172	0.3740
	S.D.	0.1342	0.1266	0.1613	0.2550	0.2404	0.2194
	MAE + 3·S.D.	0.6279	0.5778	0.7261	1.1385	1.0385	1.0322
Distribution of prediction errors (in %)	ωN in range 0.10 × Δlgk_7 (TR)_	6.6667 ^a^	6.6667 ^a^	6.6667 ^a^	25.000 ^b^	25.000 ^b^	16.667 ^b^
	ωN in range 0.15 × Δlgk_7 (TR)_	0.0000 ^a^	0.0000 ^a^	0.0000 ^a^	0.0000 ^b^	8.3333 ^b^	0.0000 ^b^
	ωN in range 0.20 × Δlgk_7 (TR)_	0.0000 ^a^	0.0000 ^a^	0.0000 ^a^	0.0000 ^b^	0.0000 ^b^	0.0000 ^b^
	ωN in range 0.25 × Δlgk_7 (TR)_	0.0000 ^a^	0.0000 ^a^	0.0000 ^a^	0.0000 ^b^	0.0000 ^b^	0.0000 ^b^
Prediction quality	-	Good					
Systematic error presence	-	Absent					

^1^ Where R^2^, R^2^_0_, and R′^2^ are determination coefficients calculated with and without taking into account the origin; $\bar{R_{m}^{2}}$ is the averaged determination coefficient of the regression function, calculated using values of determination coefficients on the ordinate axis (R^2^_m_) and using them on the abscissa (R′^2^_m_) respectively; $\Delta R_{m}^{2}$ is the difference between R^2^_m_ and R′^2^_m_; Q^2^_F1_ and Q^2^_F2,_ are determination coefficients calculated for the compounds of test sets TS1 and TS2 taking into account the average lgk_7_ value of the compounds from training and test sets, respectively; CCC is the concordance correlation coefficient; MAE is the mean absolute error; S.D. is the standard deviation; ωΝ is the percentage of test sets TS1 and TS2, for which the prediction error is less than the interval proportional to 0.1, 0.15, 0.20, and 0.25 of Δlgk_7_ of training sets TR2 (a) and TR3 (b).

**Table 6:** The lgk_7_ values of *N*-2-ethylhexyl-*N*’-phenyl-*n*-phenylendiamine (8-PPDA) predicted with the M3, M6 and M9 models.

Model	Applicability (AD)	Predicted Value lgk_7_^pred^	Predicted value k_7_^pred^·10^−5^ (L·mol^−1^·s^−1^)
M3	in AD	5.3258	2.12
M6	in AD	5.3252	2.12
M9	in AD	5.5896	3.88

**Table 7:** The dependence of the initial rate of the ethylbenzene oxidation on the 8-PPDA concentration (V_i_ = 2.4·10^−7^ mol·L^−1^·s^−1^, T = 348 K).

[8-PPDA]·10^4^ (M)	V_0_·10^6^ (mol·L^−1^·s^−1^)
0.000	4.700
0.200	2.480
0.403	0.801
0.805	0.481
1.610	0.191

## Data Availability

The structures of the compounds presented in this study are available upon request from the respective author.

## Supplementary Materials

Supplementary file contains Figures S1–S10 and Tables S1–S9.
