# Supplementary material for: QSAR Assessing the Efficiency of Antioxidants in the Termination of Radical-Chain Oxidation Processes of Organic Compounds
Source: Molecules. 2021 Jan 14;26(2):421. doi: 10.3390/molecules26020421 (PMC7830365; doi:10.3390/molecules26020421)
Supplement: Supplementary file 1 [file molecules-26-00421-s001.pdf]

QSAR assessing the efficiency of antioxidants in the termination of radical-chain oxidation processes of organic compounds

Veronika R. Khayrullina, Irina V. Safarova, Gulnaz M. Sharipova, Yuliya Z. Martynova, Anatoly Ya. Gerchikov

✉Veronika R. Khairullina

Veronika1979@yandex.ru

Bashkir State University, 32 Z. Validi str., Ufa, 450076, Russian Federation

SupplementaryMaterial

CONTENTS

1. KINETIC CLASSIFICATION OF ANTIOXIDANTS DEPENDING ON THE DEACTIVATION MODE OF OXIDATION.......... 3

2. PARAMETERS FOR ASSESSING THE DESCRIPTIVE AND PREDICTIVE POTENTIAL OF QSAR MODELS.......... 4

3. BRIEFDESCRIPTION OF THE PROGRAM GUSAR 2013 7

3.1. CALCULATION OF STRUCTURAL DESCRIPTORS 7
3.2. SELECTION OF THE DESCRIPTORS WHEN CONSRTUCTING QSAR MODELS 11
3.3. CONSTRUCTING OF THE QSAR MODELS 13
3.4. ASSESSMENT OF THE RANGE OF THE APPLICABILITY 14
4. RESULTS.......... 16

1. KINETIC CLASSIFICATION OF ANTIOXIDANTS DEPENDING ON THE DEACTIVATION MODE OF OXIDATION

1. Antioxidants terminating the chains by their reactions with peroxyl radicals (phenols, naphthols, hydroquinones, aromatic amines, aminophenols, diamines) resulting in the formation of radical intermediates with low activity.

2. Antioxidants terminating the chains by their reactions with alkyl radicals (quinones, nitrones, iminoquinones, methylenequinones, stable nitroxyl radicals, and nitro compounds). Such antioxidants are efficient at very low concentrations of dioxygen and in solid polymers).

3. Antioxidants decomposing hydroperoxide (sulfides, phosphites, arsenites, thiophosphates, carbamates, and some metal complexes) without forming free radicals. Reactions with hydroperoxides can be either stoichiometric (e.g., with sulfides and phosphites) or catalytic (e.g., chelate metal complexes).

4. Metal-deactivating antioxidants (diamines, hydroxy acids, and other bifunctional compounds) interacting with metal ions and forming the complexes inactive towards hydroperoxides.

5. Cyclic chain termination by antioxidants (aromatic amines, nitroxyl radicals, and variable-valence metal compounds).

6. Inhibitors with combined action. Such a mechanism is realized when (1) the inhibitor molecule has two and more functional groups undergoing their own reaction; and (2) the original inhibitor and its products of its transformation possess the inhibitory activities through different inhibition modes (e.g., the phenolic group of phenol sulfide reacts with peroxyl radical whereas its sulfide group is reactive towards hydroperoxide).

7. Synergetic inhibition is implemented when two inhibitors mutually enhance their inhibitory effects (e.g., in the case of 'phenol + sulfide' mixtures, in which phenol reacts with the peroxyl radical and sulfide reduces the degenerate chain branching by non-radical decomposition of hydroperoxide).

In the aspect above, a quantitative study of the antioxidant properties of natural and synthetic substances in various model systems is an important task. Assessing the antioxidant activity of individual substances and compositions may be performed with various physicochemical and biochemical methods is used [18–20]. This can be done according to their influence on the oxygen absorption (lipid peroxidation, aromatic hydrocarbons, secondary and tertiary alcohols, oxidation of crocin, chemiluminescence with luminol, oxidation of R-phycoerythrin, sensitivity of erythrocytes to hemolysis, recovery of the activity of iron ions, lipid peroxides). Some authors measure the antioxidant activity of enzymes, e.g., ascorbate-

peroxidase, glutathione reductase, dehydroascorbate reductase and mono-dehydroascorbate reductase. Herewith, in some cases, the antioxidant status of the organism correlates with the intensity of the pathology, e.g., the growth of malignant tumor cells MK-1.

Despite the diverse photometric, chromatographic and electrochemical methods, a study of the antioxidant activity (AOA) of individual compounds usually starts from the methods of chemical kinetics. In these methods, AOA compounds are involved to the model reactions such as oxidation of aliphatic and alkyl-aromatic hydrocarbons, fatty acid esters. Here, the antioxidant efficacy is estimated by the duration and depth of the inhibition of oxidation of model substrates. The main advantages of the kinetic methods for the AOA assessment are their accessibility, possibility of standardization of the substrates and the oxidation regime. The mentioned features are necessary for the reproducibility of the results [14]. A strict kinetic description of the oxidation processes and measurement of the corresponding rate constants of the elementary stages are the main advantages of this approach. However, it should be noted that the kinetic methods are non-selective to a specific antioxidant when studying the antioxidant properties of extractive compositions and mixtures of biologically active substances with a pronounced antioxidant effect [18].

2. PARAMETERS FOR ASSESSING THE DESCRIPTIVE AND PREDICTIVE POTENTIAL OF QSAR MODELS

Table S1. The equations for assessing the descriptive and predictive potentials of the QSAR models based on the R^2 and MAE metrics

Comment	Equation of the criterion	
Parameters for assessing the descriptive and predictive potential of QSAR models using internal cross-validation techniques		
Determination coefficient (Coefficient of multiple determination R^2) is the determination coefficient of the calculated using the experimental and the predicted data of the training set	$R^2 = 1 - \frac{\sum_{i=1}^{N_{TRi}} (y_i^{pred} - y_i^{obs})^2}{\sum_{i=1}^{N_{TRi}} (y_i^{obs} - \overline{y^{obs}})^2} = 1 - \frac{RSS}{TSS}$ $R^2 = \left(\frac{\sum_{i=1}^{N_{TRi}} (y_i^{obs} - \overline{y^{obs}})(y_i^{pred} - \overline{y^{pred}})}{\sqrt{\sum_{i=1}^{N_{TRi}} (y_i^{obs} - \overline{y^{obs}})^2 \times \sum_{i=1}^{N_{TRi}} (y_i^{pred} - \overline{y^{pred}})^2}} \right)^2$	(1)

R_0^2 and $R_0'^2$ are respectively the determination coefficients of the calculated using the experimental and the predicted data of the training set, forcing respectively the origin of the axis	$R_0^2 = 1 - \frac{\sum_{i=1}^{N_{TRi}} (y_i^{pred} - k \bullet y_i^{pred})^2}{\sum_{i=1}^{N_{TRi}} (y_i^{pred} - \overline{y^{pred}})^2}$ $R_0'^2 = 1 - \frac{\sum_{i=1}^{N_{TRi}} (y_i^{obs} - k' \bullet y_i^{obs})^2}{\sum_{i=1}^{N_{TRi}} (y_i^{obs} - \overline{y^{obs}})^2}$ $k = \frac{\sum_{i=1}^{N_{TRi}} (y_i^{obs} \text{晊}_i^{pred})}{\sum_{i=1}^{N_{TRi}} (y_i^{pred})^2}$; $k' = \frac{\sum_{i=1}^{N_{TRi}} (y_i^{obs} \text{晊}_i^{pred})}{\sum_{i=1}^{N_{TRi}} (y_i^{pred})^2}$	(2)
R^2_m is determination coefficient of the regression function, calculated using the experimental values on the ordinate axis, R'^2_m using them on the abscissa	$R_m^2 = R_{TRi}^2 (1 - \sqrt{R_{TRi}^2 - R_{0/TRi}^2}) > 0.5$ $\Delta R_m^2 = \lfloor R_m^2 - R_m'^2 \rfloor < 0.2$ $\overline{R_m^2} = \frac{R_m^2 + R_m'^2}{2}$	(3)
Determination coefficient by internal cross-validation	$Q^2 = Q^2_{20\%(n=20)} = 1 - \frac{\sum_{i=1}^{N_{TRi}} (y_{i/i}^{pred} - y_i^{obs})^2}{\sum_{i=1}^{N_{TRi}} (y_i^{obs} - \overline{y^{obs}})^2} = 1 - \frac{PRESS}{TSS}$	(4)
Standart deviation	$S.D. = \sqrt{\frac{\sum_{i=1}^{N_{TRi}} (y_i^{obs} - y_i^{pred})^2}{N_{TRi} - V - 1}} = \sqrt{\frac{RSS}{N_{TRi} - V - 1}}$	(5)
Root Mean Square Error in prediction activity for training set	$RMSE = \sqrt{\frac{\sum_{i=1}^{N_{TRi}} (y_i^{obs} - y_i^{pred})^2}{N_{TRi}}} = \sqrt{\frac{RSS}{N_{TRi}}}$	(6)
Variance ratio (F)	$F = \frac{\sum_{i=1}^{N_{TRi}} (y_i^{pred} - \overline{y^{obs}})^2}{\sum_{i=1}^{N_{TRi}} (y_i^{obs} - y_i^{pred})^2} \times \frac{N_{TRi} - V - 1}{V}$	(7)
Parameters of assessing the descriptive and predictive abilities of QSAR models within the external cross-validation techniques		
R_0^2 and $R_0'^2$ are calculated forcing the regression line to pass through the origin,	$R_0^2 = 1 - \frac{\sum_{i=1}^{N_{TSi}} (y_i^{pred} - k \bullet y_i^{pred})^2}{\sum_{i=1}^{N_{TSi}} (y_i^{pred} - \overline{y^{pred}})^2}$	(8)

k and k' are the slope of the regression lines	$R_0'^2 = 1 - \frac{\sum_{i=1}^{N_{TSi}} (y_i^{obs} - k' \bullet y_i^{obs})^2}{\sum_{i=1}^{N_{TSi}} (y_i^{obs} - \overline{y^{obs}})^2}$ $k = \frac{\sum_{i=1}^{N_{TSi}} (y_i^{obs} \text{旺} y_i^{pred})}{\sum_{i=1}^{N_{TSi}} (y_i^{pred})^2}$; $k' = \frac{\sum_{i=1}^{N_{TSi}} (y_i^{obs} \text{旺} y_i^{pred})}{\sum_{i=1}^{N_{TSi}} (y_i^{pred})^2}$	
Correlation coefficient between observed and predicted activities	$R_{TSi}^2 = 1 - \frac{\sum_{i=1}^{N_{TSi}} (y_i^{pred} - y_i^{obs})^2}{\sum_{i=1}^{N_{TSi}} (y_i^{obs} - \overline{y^{obs}})^2} = 1 - \frac{PRESS}{TSS}$ $R_{TSi}^2 = \left(\frac{\sum_{i=1}^{N_{TSi}} (y_i^{obs} - \overline{y^{obs}})(y_i^{pred} - \overline{y^{pred}})}{\sqrt{\sum_{i=1}^{N_{TSi}} (y_i^{obs} - \overline{y^{obs}})^2 \times \sum_{i=1}^{N_{TSi}} (y_i^{pred} - \overline{y^{pred}})^2}} \right)^2$	(9)
Determination coefficients calculated for compounds of test set TS_i, taking into account lgk_7 for compounds of the training set and average lgk_7 for compound of the test set	$Q_{F1}^2 = 1 - \frac{\sum_{i=1}^{N_{TSi}} (y_i^{pred} - y_i^{obs})^2}{\sum_{i=1}^{N_{TSi}} (y_i^{obs} - \overline{y_{i/TRi}^{obs}})^2} = 1 - \frac{PRESS}{TSS_{test}(\overline{y_{i/TRi}^{obs}})}$	(10)
	$Q_{F2}^2 = 1 - \frac{\sum_{i=1}^{N_{TSi}} (y_i^{pred} - y_i^{obs})^2}{\sum_{i=1}^{N_{TSi}} (y_i^{obs} - \overline{y_{i/TSi}^{obs}})^2} = 1 - \frac{PRESS}{TSS_{test}(\overline{y_{i/TSi}^{obs}})} = R_{TSi}^2$	(11)
Concordance Correlation Coefficient (CCC)	$CCC = \frac{2\sum_{i=1}^{N_{TSi}} (y_i^{obs} - \overline{y^{obs}})(y_i^{pred} - \overline{y^{pred}})}{\sum_{i=1}^{N_{TSi}} (y_i^{obs} - \overline{y^{obs}})^2 + \sum_{i=1}^{N_{TSi}} (y_i^{pred} - \overline{y^{pred}})^2 + N_{TSi}(\overline{y^{obs}} - \overline{y^{pred}})}$	(12)
R^2_m is determination coefficient of the regression function, calculated using the experimental values on the ordinate axis, R'^2_m using them on the abscissa	$R_m^2 = R_{TSi}^2(1 - \sqrt{R_{TSi}^2 - R_{0TSi}^2} > 0.5$ $\Delta R_m^2 = \lfloor R_m^2 - R_m'^2 \rfloor < 0.2$ $\overline{R_m^2} = \frac{R_m^2 + R_m'^2}{2}$	(13)

Root Mean Square Error in prediction activity for test set	$RMSEP = \sqrt{\frac{\sum_{i=1}^{N_{TSi}}(y_i^{obs} - y_i^{pred})^2}{N_{TSi}}} = \sqrt{\frac{RSS}{N_{TSi}}}$	(14)
Mean Absolute Error	$MAE = \frac{\sum_{i=1}^{N_{TSi}}\left\|y_i^{obs} - y_i^{pred}\right\|}{N_{TSi}}$	(15)

where

TRi is the training set, TSi is the test set,

N_{TRi} and N_{TSi} are total number of objects in the training set and test set respectively;

y_i^{obs} are experimental data values, y_i^{pred} are predicted data values;

$\overline{y^{obs}}$ are average of the experimental data values;

$\overline{y^{pred}}$ are average of the predicted data values;

RSS is residual sum of squares;

PRESS is the sum of the squares of the prediction errors (predictive sum of squares);

TSS is the total sum of squares (is sum of squared deviations from the data set mean);

$TSS_{test}(\overline{y_{i/train}^{obs}})$ and $TSS_{test}(\overline{y_{i/test}^{obs}})$ are the total sum of squares of the external set calculated using the training set mean and external set mean, respectively.

3. BRIEFDESCRIPTION OF THE PROGRAM GUSAR 2013

3.1. CALCULATION OF STRUCTURAL DESCRIPTORS

Here is a description of the GUSAR program necessary to understand the text of the article.

A detailed description of the ideology of calculating descriptors and constructing QSAR models using this program is given in the articles listed in the list of references and in the site http://www.pharmaexpert.ru (http://www.pharmaexpert.ru/passonline/downloads/articles/Filimonov-and-Poroikov-Chapter-6.pdf).

From a general point of view, the assessment of the activity of an organic molecule in the GUSAR2013 program is carried out according to the equation (1):

$$y_{pred} = a_0 + \sum_i a_i f_i(S), \qquad (1)$$

where a_0, a_1,… different functions of organic molecule’s structure S.

In classic QSAR methods, the functions $f_1(S)$, $f_2(S)$, … represent physical-chemical parameters or other quantitative characteristics of molecular structure, and the coefficients a0, a1,... are determined using multiple linear regression (MLR), partial least squares (PLS) analysis, or support vector regression (SVR), etc. [1]. QSAR methods based on the similarity between a certain molecule S_i with known biological activity and the molecule S use the value fiðSÞ of their similarity.

In the GUSAR 2013 program, the description of the structure and the calculation of the regression coefficients for the further construction of QSAR models is based on the use of two types of substructural descriptors of atomic neighborhoods: MNA (Multilevel Neighborhoods of Atoms) and QNA (Quantitative Neighborhoods of Atoms) [39, 40]. They are automatically deduced from the matrices of molecular connectivity, standard ionization potentials (IP) and electron affinities (EA). The QNA descriptors are defined by two functions, P and Q. The P and Q values for each atom *i* are calculated using the following formulae [39]:

$$P_i = B_i \sum_k \left(\exp\left(-\frac{1}{2} C \right) \right)_{ik} B_k \qquad (2)$$

$$Q_i = B_i \sum_k \left(\exp\left(-\frac{1}{2} C \right) \right)_{ik} B_k A_k \qquad (3)$$

$$A_k = \frac{1}{2}(IP_k + EA_k), \; B_k = (IP_k - EA_k)^{-1/2} \qquad (4)$$

where *k* is the remaining atoms in the molecule, IP is the first ionization potential, EA is the electron affinity for each atom (in eV), and C is the connectivity matrix for the molecule as a whole [46]. The standard values IP and EA of atoms in a molecule were collected from the literature. Although the value μP-Q can be considered by convention as the partial atomic charge, where μ is the chemical potential, in general the P and Q values are not the estimate of partial atomic charges or hardness, etc.

Any atom influences the others, although the influence decreases with the increase of the distance between them. The algorithm of the QNA descriptor calculation is really very simple due to the uselessness of the matrix Exp(-1/2C) itself, the fact that the product of Exp(-1/2C) by a vectoris needed only, and the fact that the matrix C consists of 0 and 1 only. A detailed description of QNA descriptors is represented in [45].

Thus, the QNA descriptors are calculated taking into account the relationships between all atoms of the structure. These values describe each atom of the molecule but, at the same time, depend on the structure of the molecule as a whole [45, 46]. In the future, based on the functions P and Q, the $f_i(S)$ functions are calculated. Each function of the structure of the

molecule fi (S) is calculated according to equation (4) as the average value of the function gi (P, Q) for those m atoms of the molecule that have two or more immediate neighbors:

$$f_i(S) = \frac{1}{m}\sum_k g_i(P_k, Q_k) \qquad (5)$$

Substitution of expression (5) into equation (1) and permutation of the sums allows one to obtain equation (6):

$$y_{pred} = a_0 + \sum_i a_i \frac{1}{m}\sum_k g_i(P_k, Q_k) = \frac{1}{m}\sum_k \left(a_0 + \sum_i a_i g_i(P_k, Q_k)\right) \quad (6)$$

Thus, in accordance with equation (6), the estimate of the parameter y_{pred} for a molecule is the average of the predicted values for specific atoms in the molecule. Formally, QNA descriptors represent the structure of a molecule with only two descriptors (P and Q), in contrast to the many traditional descriptors used in QSAR.

However, the developers of the GUSAR program found that the P and Q values are highly correlated with each other (r = 0.903). Since the values of P and Q have different scales (standard deviations are 0.023 and 0.208, respectively), the developers of the GUSAR program carried out normalization to optimize the family of functions g_i(P, Q). Normalization was performed by calculating mean values (E_P and E_Q), standard deviations (D_P and D_Q), and correlation between P and Q values (R_{PQ}):

$$P' = \frac{P - E_p}{D_p} \quad Q' = \frac{Q - E_Q}{D_Q} \qquad (7)$$

$$u = \frac{P' + Q'}{\sqrt{2(1 + R_{PQ})}} \qquad u = \frac{P' - Q'}{\sqrt{2(1 - R_{PQ})}} \qquad (8)$$

The orthonormal U and V have zero mean, unit variance, and they are uncorrelated [45,46].

The QNA values are the basic information for calculating the Chebyshev 2D polynomials.

$$g_i(P, Q) = T_{uv}(P, Q) = \cos(u \bullet \arccos(TanH(u))) \bullet \cos(v \bullet \arccos(TANH(v))) \qquad (9)$$

where the integers u, v=0, 1, 2,...define the 2D Chebyshev polynomial degree. The final equation for estimate y_{pred} using QNA descriptors is

$$y_{pred} = \frac{1}{m}\sum_k \left(a_0 + \sum_{uv} a_{uv} T_{uv}(P_k, Q_k)\right) = a_0 + \sum_{uv} a_{uv} T_{uv}$$
$$T_{uv} = \frac{1}{m}\sum_k \left(T_{uv}(P_k, Q_k)\right) \qquad (10)$$

Thus, the regression equations constructed in the GUSAR 2013 program take into account both the specificity and physicochemical properties of each atom entering the training set [41, 43-46, 66, 67]. However, QNA descriptors cannot be physically interpreted due to the peculiarities of their calculation. In this regard, they are not explicitly displayed under calculations.

The MNA descriptors are computed using the PASS algorithm (Prediction of Activity Spectra for Substances) [39, 40], which predicts approximately 6,400 “biological activities” with an accuracy threshold of an average prediction of at least 95%. These descriptors are generated based on the structural formulae of chemical compounds without using any pre-compiled list of structural fragments [39-41, 46]. The authors of the GUSAR 2013 program report that “MNA-descriptors are based on the molecular structure representation, which includes hydrogens according to the valences and partial charges of other atoms and does not specify the types of bonds.” They are generated as “a recursively defined sequence:

• zero-level MNA descriptor for each atom is the mark A of the atom itself;

• any next-level MNA descriptor for the atom is the substructure notation A ($D_1D_2 \ldots D_i \ldots$), where Di is the previous-level MNA descriptor for i–th immediate neighbor of the atom A.

The neighbor descriptors $D_1D_2 \ldots D_i \ldots$ are arranged in a unique manner. This may be, for example, a lexicographic sequence. MNA descriptors are generated using an iterative procedure, which results in the formation of structural descriptors that include the first, second, etc. neighborhoods of each atom. The label contains not only information about the type of atom, but also additional information about its belonging to a cyclic or acyclic system, etc. For example, an atom that does not enter a ring is marked with a “—“.

Based on the MNA descriptors using B-statistics, calculated in the PASS program, the biological activity spectrum of a chemical compound is predicted [35, 36, 42-44].

The output of the PASS program is the probabilities of the activity (Pa) and of inactivity (Pi) of each prognostic result. The difference between these two values (Pa–Pi) for a randomly selected subset of predicted activities is used as independent variables for regression analysis in GUSAR. GUSAR2013 incorporates a PASS version that pedicts 4130 types of biological activity. The developers of the GUSAR 2013 program report that the list of predictable biological activities currently includes 501 pharmacotherapeutic effects, 3295 mechanisms of action, 57 adverse and toxic effects, 199 metabolic terms, 49 transporter proteins and 29 activities related to gene expression [46]. The average accuracy of a reliable prediction of biological activity, calculated by leave-one-out cross-validation procedure is approximately

95% [68]. However, the regression equation constructed based on the MNA descriptors reveals the specificity of the action of the compound but does not explicitly reflects the physicochemical parameters of chemical compounds [46].

In addition, the GUSAR 2013 program calculates the QSAR descriptors of an entire molecule such as topological length, topological volume, lipophilicity, and physicochemical descriptors (numbers of positive and negative charges, number of donors and acceptors of the hydrogen bond, number of aromatic atoms, molecular weight and number of halogen atoms) [39, 40]. Therefore, these parameters were added to the QNA descriptors. The topological length of a molecule was calculated as the maximal distance between any two atoms and the volume of a molecule as the sum of each atom's volume, $4/3\pi R^3$, where R is the atomic radius.

The authors of the GUSAR 2013 program report that "in GUSAR, the scale of QNA- and PASS-based descriptors ranges from −1 to 1. Therefore, no additional normalization is required for these types of descriptors. Only whole-molecule descriptors are normalized using a standard Z-score normalization procedure" [40].

It should be noted that the program is able to construct QSAR models both relying solely on one of these types of descriptors, and on their combination in terms of the consensus approach [42-44]. At the same time, based on the consensus approach methodology, models for quantitative prediction of biological activity for these descriptors are calculated independently of each other. The examples of the sample QSAR GUSAR models for predicting the toxic effects of chemical compounds are available free *via* the link http://www.way2drug.com/GUSAR.

However, it noteworthy that the features of the QNA and MNA calculations retain these descriptors without unambiguous physical interpretation. For this reason, in the commercial and academic versions of the GUSAR 2013 program for broad use, the regression equations are not displayed.

3.2. SELECTION OF THE DESCRIPTORS WHEN CONSRTUCTING QSAR MODELS

In GUSAR 2013, three approaches are used when selecting the optimal number of descriptors for constructing (Q)SAR-models:

1) self-consistent regression method (SCR) [42-45];

2) method of radial basis functions (RBF) [39];

3) method based on the combination of SCR and RBF [39].

The SCR and RBF-SCR methods are the most preferable. The SCR method is correctly applied to modeling compounds with a rather high degree of similarity. The other two methods of selecting the optimal number of descriptors show good results when modeling structurally dissimilar compounds.

It was previously shown [39-44, 46, 66, 67] that self-consistent regression (SCR) can be successfully applied to various QSAR problems. The SCR method is resistant to noise in the data and allows deleting the variables that poorly describe the target value. This is a regularized method of the least squares. Independent parameters *a* are calculated in this method according to the equation (4) [43]:

$$a = \mathrm{ArgMin}\left[\sum_{i=1}^{n} y_i - \sum_{k=0}^{m} x_{ik} a_k)^2 + \sum_{k=1}^{m} v_k a_k^2\right] \qquad (4)$$

where *a* is the regression coefficient, *n* is the number of objects, y_i is the response value of the *i*-th object, *m* is the number of independent variables, x_{ik} is the value of the *k*-th independent variable of the *i*-th object, a_k is the *k*-th value of the regression coefficients, and v_k is the *k*-th value of the regularization parameters. Equation (4) hasthefollowingsolution:

$$a = TX^T y, \;\; T = (X^T X + V)^{-1}$$

where X^T is the transposed regression matrix X, and V is the diagonal matrix of the regularization parameters. The regression coefficients obtained from the SCR reflect the contribution of each particular descriptor (variable) to the final equation. The higher the absolute value of the coefficient, the greater its contribution. Thus, the regression coefficients obtained after the SCR can be used to weight the descriptors (variables) depending on their importance.

The second method used implemented in the GUSAR 2013 program for selecting the optimal number of descriptors is the interpolation method for radial basis functions RBF [39]. The authors of the GUSAR 2013 program reports [39] that, unlike the RBF network, this method uses each input variable as a center of gravity. The learning process is performed on all input variables of the training set. As can be seen from equation (5), the approximating function y(x) in the case of the RBF interpolation is represented as the sum of *N* radial basis functions, each of which is related to another center x_i and weighted by the corresponding coefficient w_i.

$$y(x) = \sum_{i=1}^{N} w_i \varphi(\|x - x_i\|) = \Phi w \qquad (5)$$

If the points x_i are different then the interpolation matrix Φ in the above equation is nonsingular. The weights w are calculated as:

$$w = \Phi^{-1} y \qquad (6)$$

Assessing the weights is based on the simple least squares method [39].

The RBF-SCR method is the third tool of the GUSAR 2013 program for selecting the optimal number of descriptors. It has a 3-step algorithm:

1) selecting descriptors using the SCR method;
2) calculating the radial basis functions using the weighted coefficient of SCR as a criterion of similarity;
3) calculating the weighting coefficients RBF by the least squares.

The RBF-SCR method can be expressed as [39]:

$$y(x) = \sum_{i=1}^{N} w_i \varphi(\|ax - a_i x_i\|) = \Phi w \qquad (7)$$

where a is taken from equation (4). Weights a_i are a new elements as compared to equation (5).

The RBF and RBF-SCR interpolation is based on a linear radial basis function that allows modeling a variety of training sets with a high level of dissimilarity between the objects.

Additionally, the GUSAR program allows visualizing the contribution of each atom into the predicted value [37-46]. This capability is implemented in the QSAR models based on the QNA descriptors and, accordingly, in the consensus combination of the QSAR models designed in different modes. It opens opportunities to identify "strong" and "weak" points in the biologically active molecules and, consequently, to rationalize the conclusions about the replacement of certain fragments upon molecular design directed to enhancing/weakening the target property.

3.3. CONSTRUCTING OF THE QSAR MODELS

The QSAR models were designed in the GUSAR 2013 program as follows. To describe the structures of compounds within the program, two types of atom-centered descriptors were used, *viz.*substructural MNA, electrotopological QNA, and, additionally, three descriptors of the whole molecule (topological length, topological volume, and lipophilicity).

The optimal set of the descriptors for constructing particular regression equations was automatically selected by the self-consistent regression [39] and sliding control procedures [37, 39, 40, 42-46]. The GUSAR 2013 program allows constructing any single QSAR models and consensus models based on them. In this study, we use the consensus approach to construct the QSAR models. This allows reducing the variability of the predictions. Consensus models were designed in GUSAR 2013 automatically based on the principle of common similarity of particular regression dependencies [37, 39-46].

The final predicted values for lgk_7 were calculated using a weighted average of the predictions from several selected QSAR models. Each model is based on a different set of QNA and MNA descriptors. Its predictions for each compound are weighted according to the similarity value as calculated during the applicability domain assessment. Note that each of these partial models involved by the consensus model was made independently based on either QNA or MNA descriptors. As a result, 9 consensus QSAR models were designed. These models included 140 partial models. However, not all of them had acceptable statistical parameters. To select the most predictive models, a 20-fold crosscheck was performed for each model. These models have the R^2 values exceed 0.6 (from the cross-validation procedure after the randomized rejection of 20% of the training set). Each of the final consensus models M1–M2, M4–M5, M7-M8 is made up with 20 particular regression dependencies. Consensus models M3, M6 and M9 include 100 regression equations. However, as the QNA and MNA descriptors have no direct physical meaning, the regression equations constructed on their basis are not explicitly displayed in the GUSAR 2013 program. Only the QSAR models satisfying the abovementioned condition have been further used for numerical predicting lgk_7 for the compounds of the external training set.

3.4. ASSESSMENT OF THE RANGE OF THE APPLICABILITY

To assess the applicability of models, GUSAR 2013 provides three different approaches based on similarity, leverage, and accuracy previously described in detail [43, 46].

Similarity. Using the Pearson correlation coefficients for each compound, we calculated the distances toward its nearest neighbors in the training set in the space of independent variables obtained after SCR. The compound is considered in the range of the model's applicability if the average value of these three distances is lower or equal to 0.7.

Leverage. The calculation of leverage allows estimating the contribution of each molecule to its own predicted value [43, 46]:

$$\text{Leverage} = x^T\left(\mathbf{X}^\mathbf{T}\mathbf{X}\right)^{-1}x,$$

where x is the vector of descriptors of the tested compound and $\mathbf{X}$ is the matrix made up with rows corresponding to the descriptors of all the molecules of the training set [43]. The compound is considered out of the applicability range if its leverage is larger than 99 % in the distribution of the leverage values of the training set.

Accuracy degree (AD). Here, the prediction of the applicability range for each compound is calculated based on the prediction error for the three most similar compounds in the test set relative to the training set as a whole [43, 46]:

$$\mathrm{AD}_{value} = \mathrm{RMSE}_{3NN} / \mathrm{RMSE}_{train}$$

In the present study, a threshold value of 1 was used for AD.

4. RESULTS

Table S2. Experimental data and values of descriptors for the phenol, aminophenol and uracil derivatives

General structural formula	Code	R_1	R_2	R_3	R_4	R_5	k_7^{obs}	lgk_7^{obs}
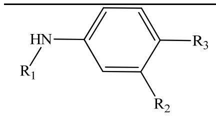	AO1	–H	$–NH_2$	–H	–	–	$8.91 \cdot 10^2$	2.95 [27]
	AO2	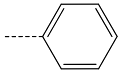	–H	–H	–	–	$4.37 \cdot 10^4$	4.64 [27]
	AO3	$–CH_3$	$–CH_3$	–H	–	–	$5.01 \cdot 10^3$	3.70 [27]
	AO4	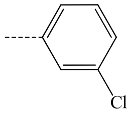	–H	–H	–	–	$1.82 \cdot 10^4$	4.26 [27]
	AO5	$–CH_3$	–H	–H	–	–	$3.98 \cdot 10^3$	3.60 [27]
	AO6	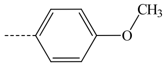	–H	$–OCH_3$	–	–	$3.31 \cdot 10^5$	5.52 [27]
	AO7	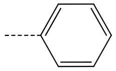	–H	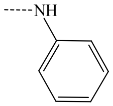	–	–	$1.70 \cdot 10^6$	6.23 [27]
	AO8	$–CH_3$	–H	$–CH_3$	–	–	$1.20 \cdot 10^4$	4.08 [27]
	AO9	$–CH_3$	–H	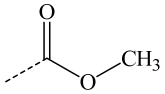	–	–	$9.12 \cdot 10^2$	2.96 [27]
	AO10	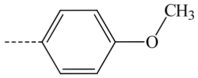	–H	–H	–	–	$2.00 \cdot 10^5$	5.30 [27]
	AO11	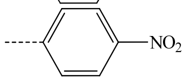	–H	–H	–	–	$6.03 \cdot 10^3$	3.78 [27]
	AO12	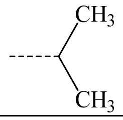	–H	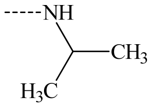	–	–	$4.47 \cdot 10^6$	6.65 [27]
	AO13	$–NH_2$	–H	–	–	–	$1.32 \cdot 10^4$	4.12 [27]
	AO14	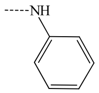	–H	–	–	–	$1.41 \cdot 10^5$	5.15 [27]

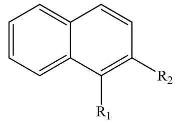	AO15	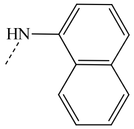	–H	–	–	–	$1.00 \cdot 10^{6}$	6.00 [27]
	AO16	–H	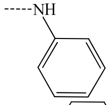	–	–	–	$1.00 \cdot 10^{5}$	5.00 [27]
	AO17	–H	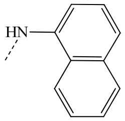	–	–	–	$1.82 \cdot 10^{5}$	5.26 [27]
	AO18	–H	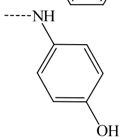	–	–	–	$1.58 \cdot 10^{6}$	6.20 [27]
	AO19	–H	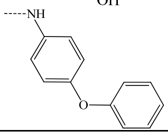	–	–	–	$4.79 \cdot 10^{5}$	5.68 [27]
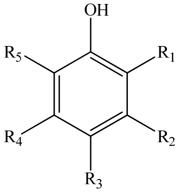	AO20	–H	–H	–H	–H	–H	$3.02 \cdot 10^{3}$	3.48 [27]
	AO21	–H	–H	$-OCH_3$	–H	–H	$4.79 \cdot 10^{4}$	4.68 [27]
	AO22	–H	–H	$-CH_3$	–H	–H	$2.00 \cdot 10^{4}$	4.30 [27]
	AO23	–H	–H	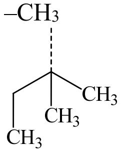	–H	–H	$1.62 \cdot 10^{4}$	4.21 [27]
	AO24	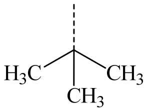	–H	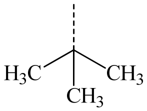	–H	–H	$1.91 \cdot 10^{4}$	4.28 [27]
	AO25	–H	–H	–Cl	–H	–H	$4.68 \cdot 10^{3}$	3.67 [27]
	AO26	–H	$-OCH_3$	–H	–H	–H	$5.75 \cdot 10^{3}$	3.76 [27]
	AO27	–H	–Cl	–H	–H	–H	$1.23 \cdot 10^{4}$	4.09 [27]
	AO28	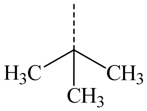	–H	–H	–H	–H	$1.66 \cdot 10^{4}$	4.22 [27]
	AO29	–H	$-CH_3$	–H	–H	–H	$2.40 \cdot 10^{4}$	4.38 [27]

AO30	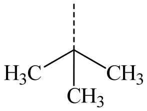	–H	–H	–H	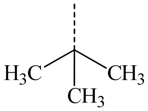	$5.01 \cdot 10^3$	3.70 [27]
AO31	$-CH_3$	–H	$-CH_3$	–H	–H	$4.07 \cdot 10^4$	4.61 [27]
AO32	$-CH_3$	$-CH_3$	–H	–H	–H	$2.00 \cdot 10^4$	4.30 [27]
AO33	$-C_2H_5$	–H	–H	–H	$-C_2H_5$	$2.19 \cdot 10^4$	4.34 [27]
AO34	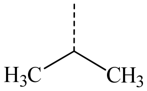	–H	–H	–H	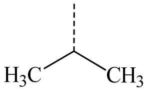	$2.09 \cdot 10^4$	4.32 [27]
AO35	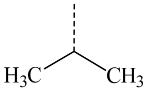	–H	$-CH_3$	–H	–H	$3.89 \cdot 10^4$	4.59 [27]
AO36	$-CH_3$	–H	–H	–H	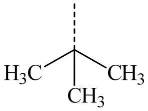	$1.58 \cdot 10^4$	4.20 [27]
AO37	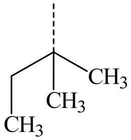	–H	$-CH_3$	–H	–H	$4.37 \cdot 10^4$	4.64 [27]
AO38	–H	$-CH_3$	$-CH_3$	–H	$-CH_3$	$4.79 \cdot 10^4$	4.68 [27]
AO39	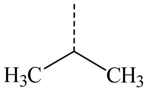	–H	$-CH_3$	–H	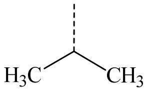	$5.75 \cdot 10^4$	4.76 [27]
AO40	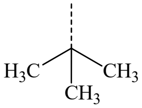	–H	$-CH_3$	–H	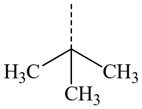	$1.00 \cdot 10^4$	4.00 [27]
AO41	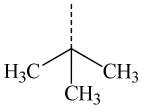	–H	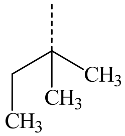	–H	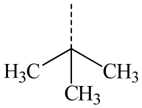	$1.58 \cdot 10^4$	4.20 [27]
AO42	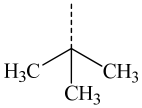	–H	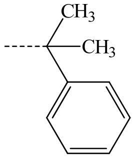	–H	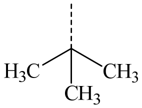	$1.35 \cdot 10^4$	4.13 [27]

AO43	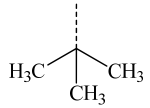	–H	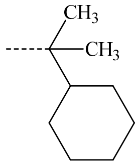	–H	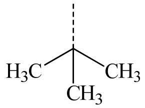	$1.45 \cdot 10^4$	4.16 [27]
AO44	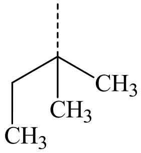	–H	$–CH_3$	–H	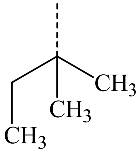	$1.58 \cdot 10^4$	4.20 [27]
AO45	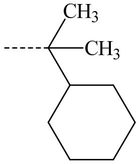	–H	$–CH_3$	–H	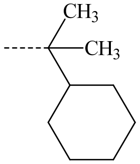	$3.63 \cdot 10^4$	4.56 [27]
AO46	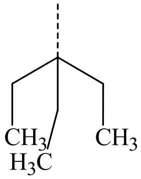	–H	$–CH_3$	–H	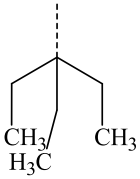	$1.58 \cdot 10^4$	4.20 [27]
AO47	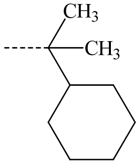	–H	$–CH_3$	–H	–H	$1.45 \cdot 10^4$	4.16 [27]
AO48	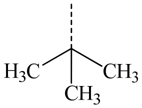	–H	$–OCH_3$	–H	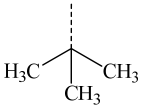	$7.76 \cdot 10^4$	4.89 [27]
AO49	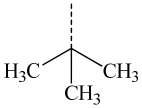	–H	–Cl	–H	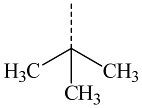	$6.76 \cdot 10^3$	3.83 [27]
AO50	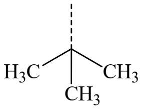	–H	–CN	–H	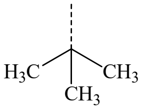	$1.70 \cdot 10^3$	3.23 [27]

AO51	–H	–H	–CN	–H	–H	$6.46\cdot10^3$	3.81 [27]
AO52	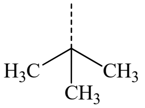	–H	$–NO_2$	–H	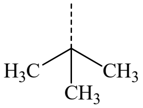	$1.00\cdot10^3$	3.00 [27]
AO53	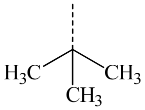	–H	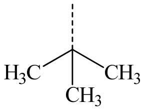	–H	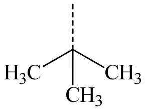	$1.62\cdot10^4$	4.21 [27]
AO54	$–CH_3$	$–CH_3$	–H	$–CH_3$	$–CH_3$	$2.95\cdot10^4$	4.47 [27]
AO55	$–CH_3$	$–CH_3$	$–CH_3$	$–CH_3$	$–CH_3$	$8.51\cdot10^4$	4.93 [27]
AO56	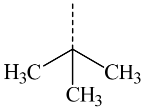	–H	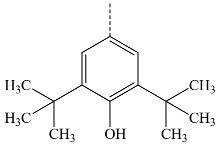	–H	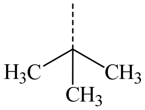	$4.17\cdot10^4$	4.62 [27]
AO57	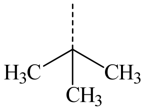	–H	–CHO	–H	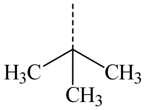	$1.78\cdot10^3$	3.25 [27]
AO58	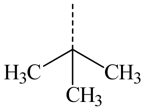	–H	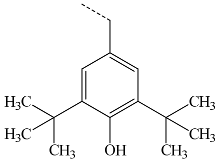	–H	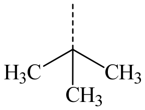	$3.02\cdot10^4$	4.48 [27]
AO59	–H	–H	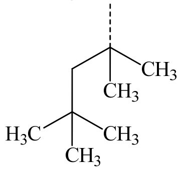	–H	–H	$3.80\cdot10^4$	4.58 [27]
AO60	$–CH_3$	–H	–H	–H	–H	$2.51\cdot10^4$	4.40 [27]
AO61	–H	–H	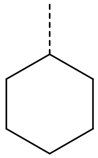	–H	–H	$3.80\cdot10^4$	4.58 [27]
AO62	$–CH_3$	–H	$–CH_3$	–H	$–CH_3$	$1.91\cdot10^5$	5.28 [27]

	AO63	–H	–H	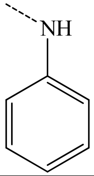	–H	–H	$1.00 \cdot 10^6$	6.00 [27]
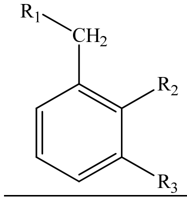	AO64	–H	–H	–H	–	–	1.26	0.10 [49]
	AO65	$–CH_3$	–H	–H	–	–	1.58	0.20 [49]
	AO66	–H	–H	$–CH_3$	–	–	1.41	0.15 [49]
	AO67	–H	$–CH_3$	–H	–	–	1.58	0.20 [49]
	AO68	–H	–H	$–C_2H_5$	–	–	1.38	0.14 [49]
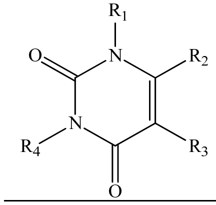	AO69	–	$–CH_3$	–OH	–H	–	$2.57 \cdot 10^4$	4.41 [48]
	AO70	–	–H	$–NH_2$	–H	–	$2.00 \cdot 10^4$	4.30 [27]
	AO71	–	$–CH_3$	–OH	$–CH_3$	–	$6.76 \cdot 10^4$	4.83 [27]
	AO72	–	–H	–OH	–H	–	$6.76 \cdot 10^3$	3.83 [27]
	AO73	$–CH_3$	$–CH_3$	$–NH_2$	$–CH_3$	–	$2.09 \cdot 10^5$	5.32 [27]
	AO74	$–CH_3$	$–CH_3$	–OH	$–CH_3$	–	$1.07 \cdot 10^4$	4.03 [27]

Table S3. Prediction of the lgk_7 values for the TR1 compounds using models M1-M3.*

Name	lgk_7^{obs}	M1		M2		M3	
		lgk_7^{pred}	$\|\Delta lgk_7\|$	lgk_7^{pred}	$\|\Delta lgk_7\|$	lgk_7^{pred}	$\|\Delta lgk_7\|$
AO 1	4.640	4.631	0.009	4.641	0.001	4.620	0.020
AO 2	6.000	5.690	0.310	5.541	0.459	5.837	0.163
AO 3	4.115	4.109	0.006	4.105	0.010	4.111	0.004
AO 4	5.150	5.146	0.004	5.155	0.005	5.145	0.005
AO 5	5.255	5.251	0.004	5.258	0.003	5.261	0.006
AO 6	5.000	4.993	0.007	5.006	0.005	5.011	0.011
AO 7	3.600	3.571	0.029	3.627	0.027	3.566	0.034
AO 8	3.700	3.648	0.052	3.650	0.050	3.645	0.055
AO 9	4.255	4.395	0.140	4.387	0.132	4.401	0.146
AO 10	2.950	3.144	0.194	3.098	0.148	3.191	0.241
AO 11	5.520	5.540	0.020	5.509	0.011	5.494	0.026
AO 12	6.230	6.012	0.218	6.148	0.082	6.585	0.355
AO 13	4.080	3.952	0.128	3.984	0.096	3.918	0.162
AO 14	2.955	3.189	0.234	3.172	0.217	3.204	0.249
AO 15	5.300	5.215	0.085	5.210	0.090	5.218	0.082
AO 16	3.780	3.982	0.202	4.015	0.235	3.947	0.167
AO 17	6.200	6.139	0.061	6.153	0.047	6.124	0.077
AO 18	5.680	5.645	0.035	5.615	0.065	5.673	0.007
AO 19	3.480	3.607	0.127	3.619	0.139	3.594	0.114
AO 20	4.680	4.596	0.084	4.597	0.083	4.596	0.084
AO 21	4.300	4.262	0.038	4.265	0.035	4.258	0.042
AO 22	4.205	4.186	0.019	4.223	0.018	4.226	0.021
AO 23	4.280	4.312	0.032	4.237	0.043	4.300	0.020
AO 24	3.670	3.775	0.105	3.768	0.098	3.783	0.113
AO 25	3.755	3.954	0.199	3.529	0.227	3.926	0.171
AO 26	4.090	4.030	0.060	4.064	0.026	3.996	0.095
AO 27	4.220	4.068	0.152	4.222	0.002	4.118	0.102
AO 28	4.380	4.297	0.083	4.308	0.072	4.286	0.094
AO 29	3.700	3.927	0.227	3.854	0.154	3.801	0.101
AO 30	4.610	4.541	0.069	4.596	0.014	4.486	0.124
AO 31	4.300	4.284	0.016	4.321	0.021	4.311	0.011
AO 32	4.335	4.273	0.062	4.260	0.075	4.286	0.049
AO 33	4.320	4.324	0.004	4.327	0.007	4.319	0.001
AO 34	4.590	4.556	0.034	4.570	0.020	4.542	0.048
AO 35	4.200	4.222	0.022	4.225	0.025	4.180	0.020
AO 36	4.640	4.438	0.202	4.525	0.115	4.550	0.090
AO 37	4.675	4.657	0.018	4.651	0.024	4.688	0.013
AO 38	4.755	4.727	0.028	4.735	0.020	4.719	0.037
AO 39	4.000	3.828	0.172	4.022	0.022	4.122	0.122
AO 40	4.200	4.087	0.113	4.219	0.019	4.208	0.008
AO 41	4.130	4.140	0.010	4.141	0.011	4.122	0.008
AO 42	4.155	4.175	0.020	4.122	0.033	4.162	0.007

AO 43	4.200	4.237	0.037	4.239	0.039	4.236	0.035
AO 44	4.560	4.566	0.006	4.549	0.011	4.560	0.000
AO 45	4.200	4.237	0.037	4.229	0.029	4.245	0.045
AO 46	4.160	4.169	0.009	4.158	0.002	4.176	0.016
AO 47	4.890	4.358	0.532	4.643	0.247	4.633	0.257
AO 48	3.830	4.027	0.197	3.891	0.061	3.963	0.133
AO 49	3.230	3.410	0.180	3.426	0.196	3.393	0.163
AO 50	3.810	3.826	0.016	3.813	0.003	3.838	0.028
AO 51	3.000	3.203	0.203	3.191	0.191	3.215	0.215
AO 52	4.205	4.011	0.194	4.216	0.011	4.029	0.176
AO 53	4.470	4.484	0.014	4.478	0.008	4.450	0.020
AO 54	4.930	4.832	0.098	4.804	0.126	4.860	0.070
AO 55	4.620	4.604	0.016	4.632	0.012	4.600	0.020
AO 56	3.245	3.267	0.022	3.359	0.114	3.394	0.149
AO 57	4.480	4.499	0.019	4.462	0.018	4.461	0.020
AO 58	4.580	4.489	0.091	4.477	0.103	4.500	0.080
AO 59	4.400	4.246	0.154	4.381	0.019	4.311	0.089
AO 60	4.580	4.523	0.057	4.581	0.001	4.467	0.113
AO 61	5.280	5.042	0.238	4.998	0.282	5.087	0.193
AO 62	6.000	5.799	0.201	5.978	0.022	5.819	0.181
AO 63	0.100	0.366	0.266	0.208	0.108	0.323	0.223
AO 64	0.200	0.393	0.193	0.402	0.202	0.384	0.184
AO 65	0.150	0.239	0.089	0.244	0.094	0.235	0.085
AO 66	0.200	0.747	0.547	0.311	0.111	0.344	0.144
AO 67	0.140	0.578	0.438	0.648	0.508	0.508	0.368
AO 68	6.650	6.091	0.559	6.164	0.486	6.258	0.392
AO 69	4.414	4.250	0.164	4.427	0.013	4.529	0.115
AO 70	5.322	5.128	0.194	5.006	0.316	5.393	0.071
AO 71	4.301	4.251	0.050	4.315	0.014	4.347	0.046
AO 72	4.029	4.293	0.264	4.159	0.130	4.228	0.199
AO 73	4.831	4.784	0.047	4.772	0.059	4.867	0.036
AO 74	3.826	3.981	0.155	4.002	0.176	3.960	0.134

* The falling outresults are marked by red.

Table S4. Prediction of the lgk_7 values for the TR2 compounds using models M4-M6.*

Name	lgk_7^{obs}	M4		M5		M6	
		lgk_7^{pred}	$\|\Delta lgk_7\|$	lgk_7^{pred}	$\|\Delta lgk_7\|$	lgk_7^{pred}	$\|\Delta lgk_7\|$
AO 1	4.640	4.457	0.183	4.567	0.073	4.646	0.006
AO 2	6.000	5.716	0.284	5.829	0.172	5.772	0.228
AO 3	4.115	4.381	0.266	4.294	0.179	4.266	0.151
AO 5	5.255	5.333	0.078	5.291	0.036	5.322	0.067
AO 6	5.000	5.195	0.195	5.276	0.276	5.195	0.195
AO 7	3.600	3.653	0.053	3.703	0.103	3.713	0.113
AO 8	3.700	3.897	0.197	3.757	0.057	3.796	0.096
AO 9	4.255	4.267	0.012	4.332	0.077	4.344	0.089
AO 10	2.950	3.024	0.074	3.018	0.068	3.083	0.133
AO 14	2.955	3.117	0.162	3.125	0.170	3.213	0.258
AO 15	5.300	4.961	0.339	5.174	0.126	5.114	0.186
AO 16	3.780	4.062	0.282	4.073	0.293	4.066	0.286
AO 17	6.200	5.991	0.209	6.011	0.189	5.971	0.229
AO 18	5.680	5.660	0.020	5.595	0.085	5.631	0.049
AO 19	3.480	3.680	0.200	3.753	0.273	3.718	0.238
AO 20	4.680	4.422	0.258	4.497	0.183	4.477	0.204
AO 23	4.280	4.232	0.049	4.334	0.053	4.299	0.019
AO 25	3.755	3.956	0.201	4.032	0.277	3.988	0.233
AO 26	4.090	4.080	0.010	4.124	0.034	4.112	0.022
AO 27	4.220	4.256	0.036	4.246	0.026	4.225	0.005
AO 29	3.700	3.770	0.070	3.849	0.149	3.819	0.119
AO 30	4.610	4.605	0.005	4.616	0.006	4.583	0.027
AO 31	4.300	4.329	0.029	4.309	0.009	4.330	0.030
AO 32	4.335	4.423	0.088	4.336	0.001	4.344	0.009
AO 33	4.320	4.382	0.062	4.375	0.055	4.362	0.042
AO 34	4.590	4.551	0.039	4.569	0.021	4.534	0.056
AO 35	4.200	4.246	0.046	4.265	0.065	4.237	0.037
AO 36	4.640	4.550	0.090	4.484	0.156	4.499	0.141
AO 37	4.675	4.663	0.012	4.685	0.010	4.666	0.009
AO 39	4.000	3.836	0.164	4.001	0.001	3.990	0.010
AO 40	4.200	4.180	0.020	4.196	0.004	4.174	0.026
AO 41	4.130	4.167	0.037	4.151	0.021	4.150	0.020
AO 42	4.155	4.158	0.003	4.167	0.012	4.156	0.001
AO 43	4.200	4.221	0.021	4.222	0.022	4.218	0.018
AO 45	4.200	4.219	0.019	4.193	0.007	4.213	0.013
AO 47	4.890	4.644	0.246	4.581	0.309	4.641	0.249
AO 48	3.830	3.858	0.028	3.856	0.026	3.882	0.051
AO 49	3.230	3.362	0.132	3.463	0.233	3.446	0.216
AO 52	4.205	4.150	0.055	4.151	0.054	4.136	0.069
AO 53	4.470	4.585	0.115	4.543	0.073	4.551	0.081
AO 54	4.930	4.806	0.124	4.804	0.126	4.800	0.130
AO 56	3.245	3.325	0.079	3.421	0.176	3.434	0.189

AO 57	4.480	4.376	0.105	4.432	0.049	4.413	0.067
AO 58	4.580	4.232	0.349	4.334	0.247	4.299	0.281
AO 59	4.400	4.291	0.109	4.269	0.131	4.333	0.067
AO 60	4.580	4.498	0.082	4.528	0.052	4.516	0.064
AO 61	5.280	5.042	0.238	5.079	0.201	5.096	0.184
AO 62	6.000	5.823	0.177	5.959	0.041	5.811	0.189
AO 63	0.100	0.100	0.000	0.100	0.000	0.235	0.135
AO 64	0.200	0.195	0.005	0.196	0.004	0.215	0.015
AO 66	0.200	0.200	0.000	0.200	0.000	0.205	0.005
AO 67	0.140	0.140	0.000	0.148	0.008	0.220	0.080
AO 68	6.650	6.277	0.373	6.383	0.267	6.254	0.396
AO 69	4.414	4.372	0.042	4.336	0.078	4.356	0.058
AO 70	5.322	5.070	0.252	5.085	0.237	5.084	0.238
AO 71	4.301	4.262	0.040	4.296	0.005	4.274	0.027
AO 72	4.029	4.139	0.110	4.206	0.177	4.225	0.196
AO 73	4.831	4.811	0.020	4.808	0.023	4.762	0.069
AO 74	3.826	4.013	0.187	3.993	0.167	3.987	0.161

* The falling outresults are marked by red.

Table S5. Prediction of the lgk_7 values for the TR3 compounds using models M7-M9.*

Name	lgk_7^{obs}	M7		M8		M9	
		lgk_7^{pred}	$\|\Delta lgk_7\|$	lgk_7^{pred}	$\|\Delta lgk_7\|$	lgk_7^{pred}	$\|\Delta lgk_7\|$
AO 1	4.640	4.573	0.067	4.632	0.008	4.676	0.036
AO 3	4.115	4.394	0.279	4.150	0.034	4.342	0.227
AO 4	5.150	5.019	0.131	4.967	0.183	5.076	0.074
AO 6	5.000	5.005	0.005	5.061	0.061	5.041	0.041
AO 7	3.600	3.737	0.137	3.688	0.088	3.755	0.155
AO 8	3.700	3.815	0.115	3.746	0.046	3.789	0.089
AO 9	4.255	4.229	0.026	4.358	0.103	4.361	0.106
AO 10	2.950	3.262	0.312	2.950	0.000	3.249	0.299
AO 11	5.520	5.272	0.248	5.403	0.117	5.321	0.199
AO 12	6.230	6.176	0.054	6.028	0.202	6.019	0.211
AO 13	4.080	4.003	0.077	3.953	0.127	3.970	0.110
AO 14	2.955	3.262	0.307	3.146	0.191	3.199	0.244
AO 15	5.300	5.038	0.263	5.269	0.031	5.185	0.116
AO 16	3.780	4.059	0.279	4.139	0.359	4.079	0.299
AO 17	6.200	6.120	0.080	6.063	0.137	6.025	0.175
AO 18	5.680	5.627	0.053	5.500	0.180	5.512	0.168
AO 19	3.480	3.668	0.188	3.756	0.276	3.678	0.198
AO 20	4.680	4.444	0.236	4.499	0.181	4.491	0.189
AO 21	4.300	4.318	0.018	4.286	0.014	4.313	0.013
AO 22	4.205	4.262	0.057	4.263	0.058	4.250	0.045
AO 23	4.280	4.233	0.047	4.335	0.055	4.278	0.002
AO 24	3.670	3.811	0.141	3.826	0.156	3.810	0.140
AO 26	4.090	4.012	0.078	4.057	0.033	4.025	0.065
AO 27	4.220	4.243	0.023	4.325	0.105	4.261	0.041
AO 28	4.380	4.372	0.008	4.347	0.033	4.358	0.022
AO 29	3.700	3.778	0.078	3.820	0.120	3.826	0.126
AO 31	4.300	4.322	0.022	4.289	0.011	4.325	0.025
AO 32	4.335	4.392	0.057	4.366	0.031	4.358	0.023
AO 33	4.320	4.387	0.067	4.359	0.039	4.364	0.044
AO 34	4.590	4.528	0.062	4.554	0.036	4.541	0.049
AO 35	4.200	4.274	0.074	4.274	0.074	4.244	0.044
AO 36	4.640	4.474	0.166	4.484	0.156	4.496	0.144
AO 37	4.675	4.730	0.055	4.681	0.006	4.695	0.020
AO 39	4.000	3.892	0.108	3.986	0.014	3.957	0.043
AO 40	4.200	4.139	0.061	4.190	0.010	4.165	0.035
AO 41	4.130	4.166	0.036	4.157	0.027	4.169	0.039
AO 42	4.155	4.181	0.026	4.196	0.041	4.194	0.039
AO 43	4.200	4.176	0.024	4.218	0.018	4.191	0.010
AO 44	4.560	4.426	0.134	4.426	0.134	4.450	0.110
AO 45	4.200	4.214	0.014	4.208	0.008	4.208	0.008
AO 47	4.890	4.761	0.129	4.641	0.249	4.668	0.222
AO 48	3.830	3.833	0.002	3.841	0.011	3.872	0.042

AO 49	3.230	3.285	0.055	3.490	0.260	3.473	0.243
AO 50	3.810	3.873	0.063	3.940	0.130	3.907	0.097
AO 51	3.000	3.262	0.262	3.385	0.385	3.281	0.281
AO 54	4.930	4.823	0.107	4.807	0.123	4.838	0.092
AO 55	4.620	4.519	0.102	4.529	0.091	4.534	0.086
AO 57	4.480	4.450	0.030	4.471	0.010	4.451	0.029
AO 58	4.580	4.233	0.347	4.335	0.245	4.278	0.302
AO 59	4.400	4.372	0.028	4.334	0.066	4.369	0.031
AO 60	4.580	4.496	0.084	4.523	0.057	4.540	0.040
AO 61	5.280	5.089	0.191	5.188	0.092	5.125	0.155
AO 62	6.000	5.977	0.023	5.967	0.033	5.868	0.133
AO 63	0.100	0.100	0.000	0.100	0.000	0.134	0.034
AO 65	0.150	0.150	0.000	0.150	0.000	0.152	0.002
AO 66	0.200	0.200	0.000	0.200	0.000	0.282	0.082
AO 67	0.140	0.140	0.000	0.140	0.000	0.140	0.000
AO 68	6.650	6.187	0.463	6.328	0.323	6.140	0.510
AO 69	4.414	4.383	0.031	4.370	0.044	4.381	0.033
AO 70	5.322	5.099	0.223	5.108	0.214	5.113	0.209
AO 73	4.831	4.735	0.096	4.802	0.029	4.811	0.021
AO 74	3.826	3.952	0.126	4.017	0.191	3.999	0.173

* The falling outresults are marked by red.

Table S6. Prediction of the lgk_7 values for the TS1 compounds using models M4-M6.*

Name	lgk_7^{obs}	M4		M5		M6	
		lgk_7^{pred}	$\lvert\Delta lgk_7\rvert$	lgk_7^{pred}	$\lvert\Delta lgk_7\rvert$	lgk_7^{pred}	$\lvert\Delta lgk_7\rvert$
AO 4	5.150	4.966	0.184	4.881	0.269	5.170	0.020
AO 11	5.520	5.130	0.390	5.376	0.144	5.206	0.314
AO 12	6.230	5.895	0.335	6.003	0.227	5.635	0.595
AO 13	4.080	4.142	0.062	3.881	0.199	3.860	0.220
AO 21	4.300	4.155	0.145	4.224	0.076	4.142	0.158
AO 22	4.205	4.423	0.218	4.275	0.070	4.332	0.127
AO 24	3.670	4.066	0.396	4.056	0.386	4.078	0.408
AO 28	4.380	4.004	0.376	4.023	0.357	4.041	0.340
AO 38	4.755	4.415	0.340	4.424	0.332	4.364	0.391
AO 44	4.560	4.584	0.024	4.599	0.039	4.532	0.028
AO 46	4.160	4.437	0.277	4.309	0.149	4.367	0.207
AO 50	3.810	3.838	0.028	4.185	0.375	4.017	0.207
AO 51	3.000	3.841	0.841	3.852	0.852	3.874	0.874
AO 55	4.620	4.502	0.118	4.679	0.059	4.545	0.075
AO 65	0.150	0.414	0.264	0.242	0.092	0.452	0.302

* The falling outresults are marked by red.

Table S7. Prediction of the lgk_7 values for the TS2 compounds using models M7-M9.*

Name	lgk_7^{obs}	M7		M8		M9	
		lgk_7^{pred}	$\lvert\Delta lgk_7\rvert$	lgk_7^{pred}	$\lvert\Delta lgk_7\rvert$	lgk_7^{pred}	$\lvert\Delta lgk_7\rvert$
AO 2	6.000	5.108	0.892	4.992	1.008	5.134	0.866
AO 5	5.255	5.296	0.041	5.251	0.004	5.278	0.023
AO 25	3.755	4.339	0.584	4.430	0.675	4.453	0.698
AO 30	4.610	4.550	0.060	4.649	0.039	4.532	0.078
AO 38	4.755	4.296	0.459	4.322	0.433	4.314	0.441
AO 46	4.160	4.395	0.235	4.287	0.127	4.351	0.191
AO 52	4.205	3.909	0.296	3.757	0.448	3.942	0.263
AO 53	4.470	4.840	0.370	4.624	0.154	4.770	0.300
AO 56	3.245	3.945	0.700	3.958	0.713	3.840	0.595
AO 64	0.200	0.280	0.080	0.373	0.173	0.708	0.508
AO 71	4.301	3.541	0.760	3.928	0.373	3.851	0.450
AO 72	4.029	4.553	0.524	4.382	0.353	4.596	0.567

* The falling outresults are marked by red.

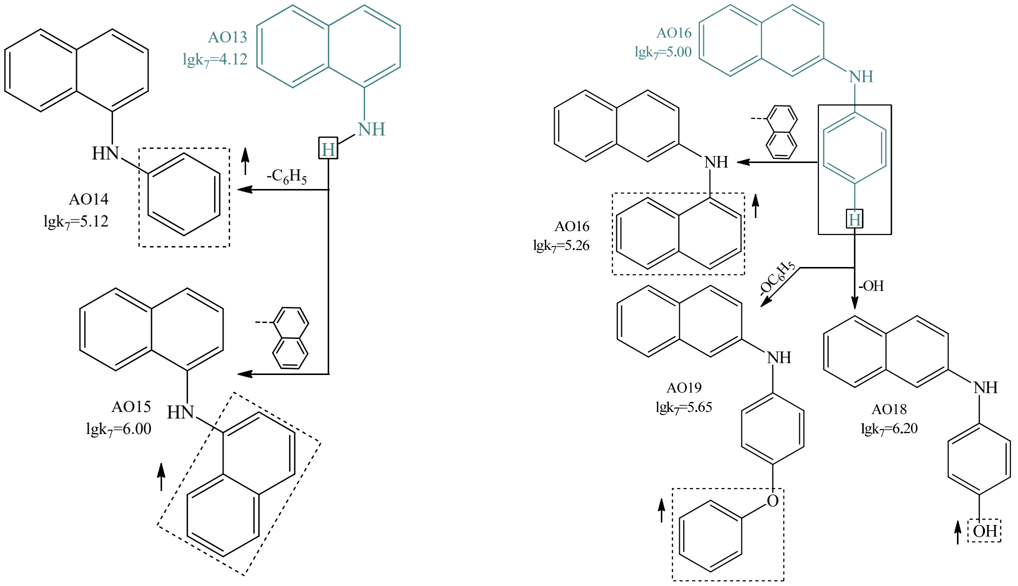

Figure S1. The effect of structural features on the antioxidant activity of compounds with the general structural formula II.

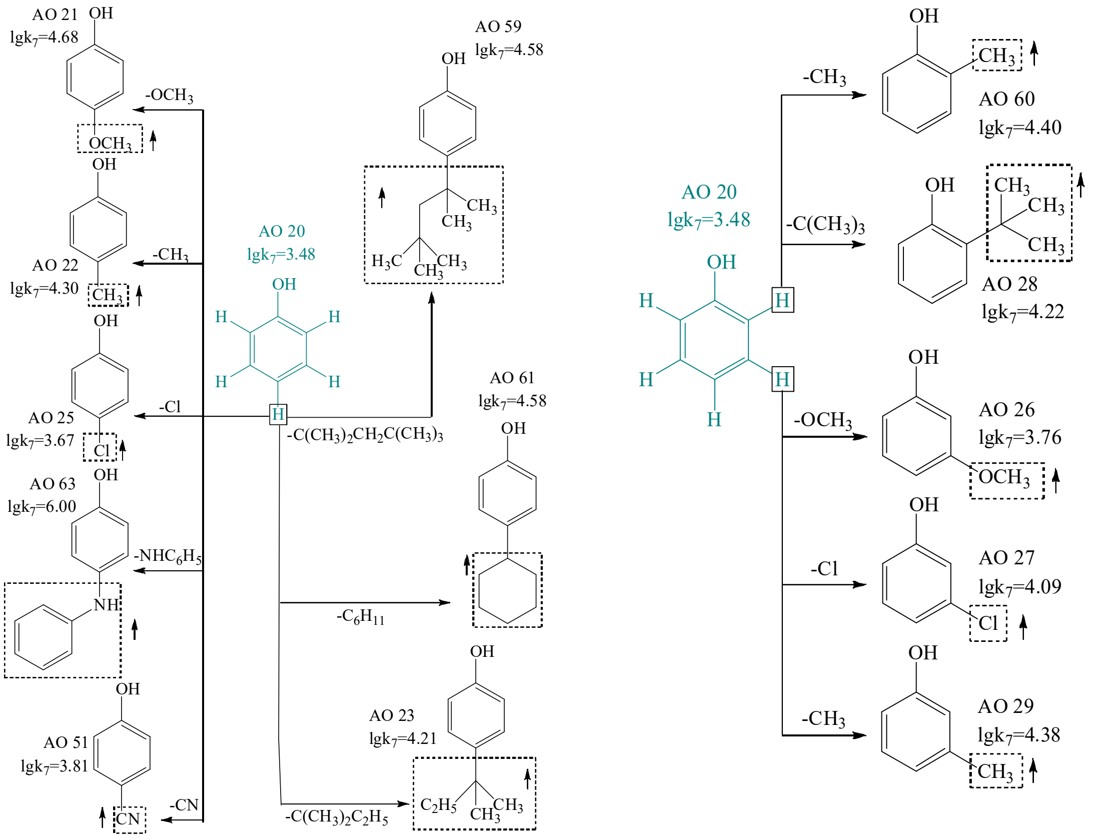

a) b)

Figure S2. Influence of para (a), ortho and meta (b) substituents on the antioxidant activity of compounds III.

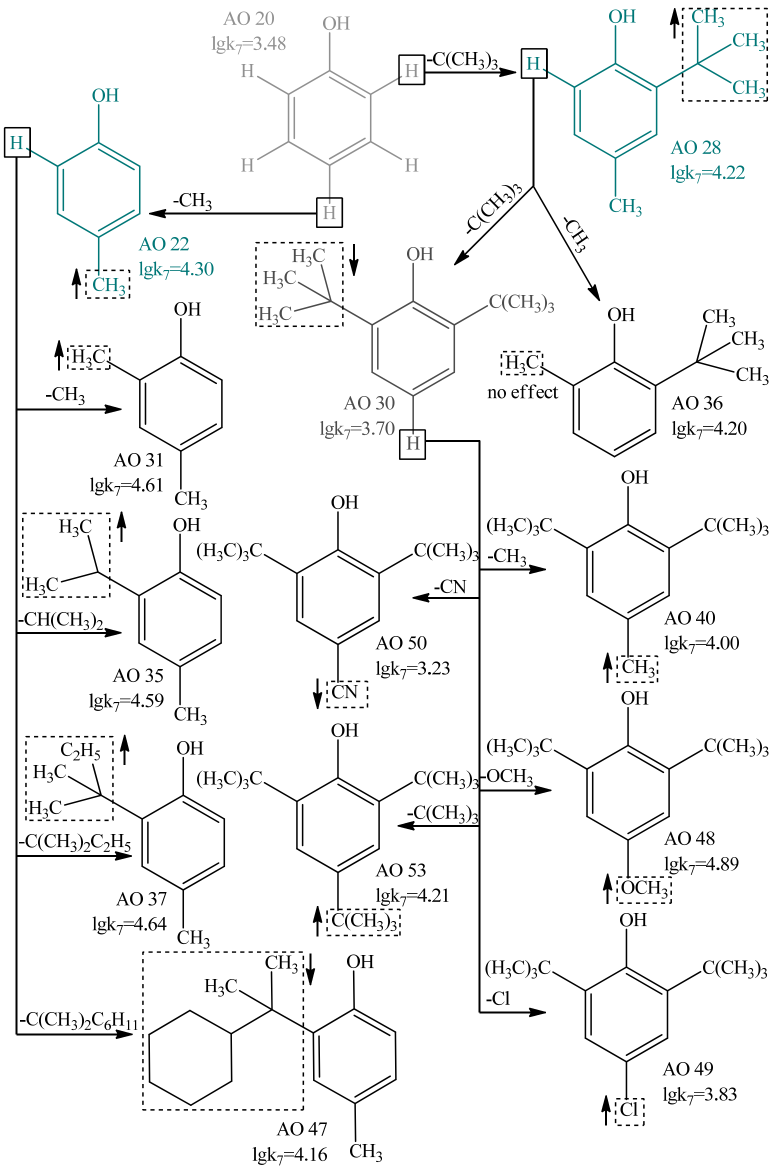

Figure S3. Influence of ortho and para substituents on the antioxidant activity of compounds III.

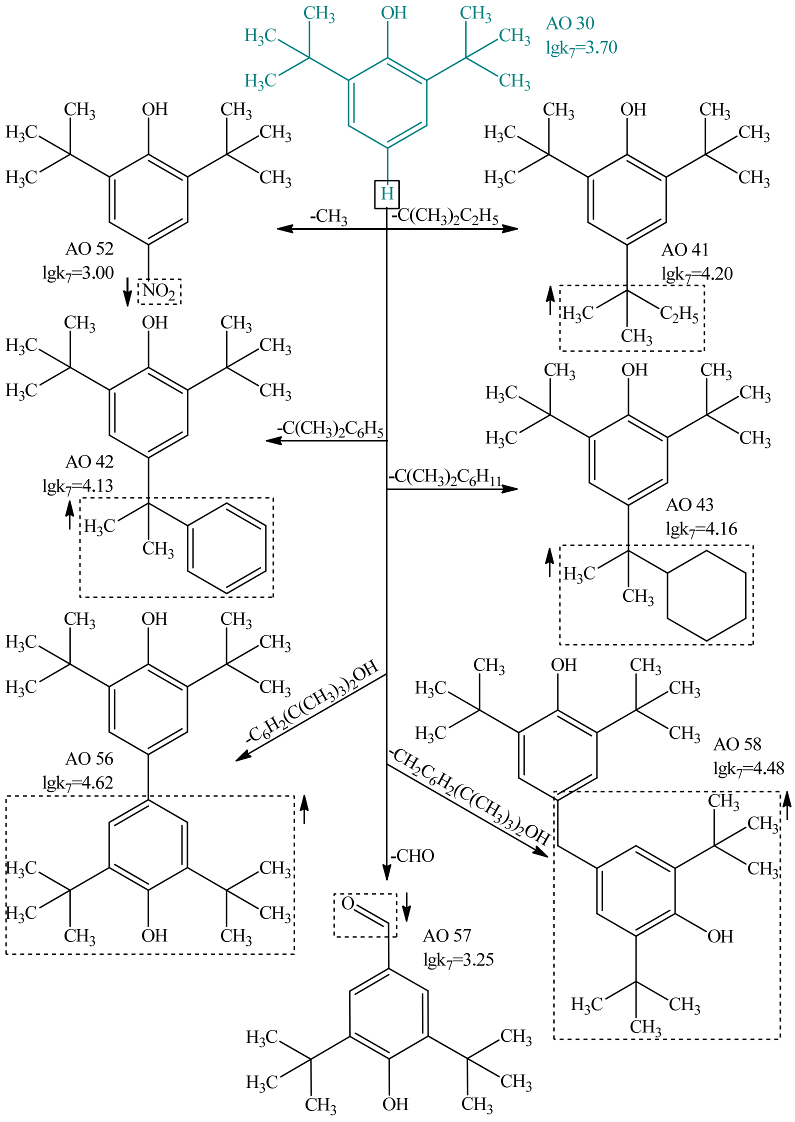

Figure S4. Effect of para substituents on the antioxidant activity of compounds III with two tert-butyl fragments in the ortho-position.

Analyzing the amine and phenol compounds I-III, we have revealed the para-substituent effects of benzene ring on the antioxidant activity. The lgk_7 values of para-substituted phenols strongly depend on the resonant effect of para-substituents. The functional groups with positive resonant effect increase lgk_7. Such increases is known in physical organic chemistry and explained with the increase in the conjugation in the aromatic system due to the presence of the functional groups with positive mesomeric effect [28].

Herewith, the field and inductive effects do not influence pronouncedly on AOA of phenols with para-substituents [29]. The meta-substituent effects were not considered due to the lack of such structures in the training sets.

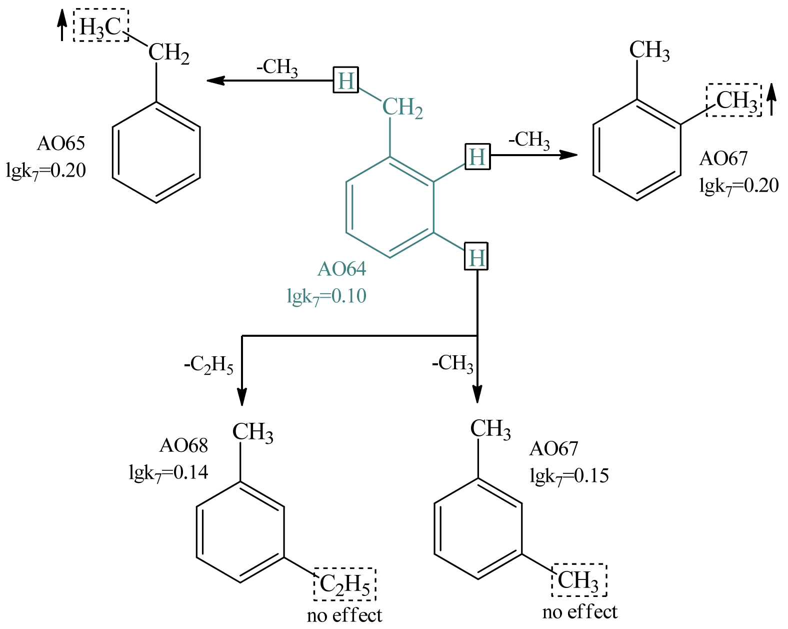

Figure 5S. The effect of structural features on the antioxidant activity of compounds IV.

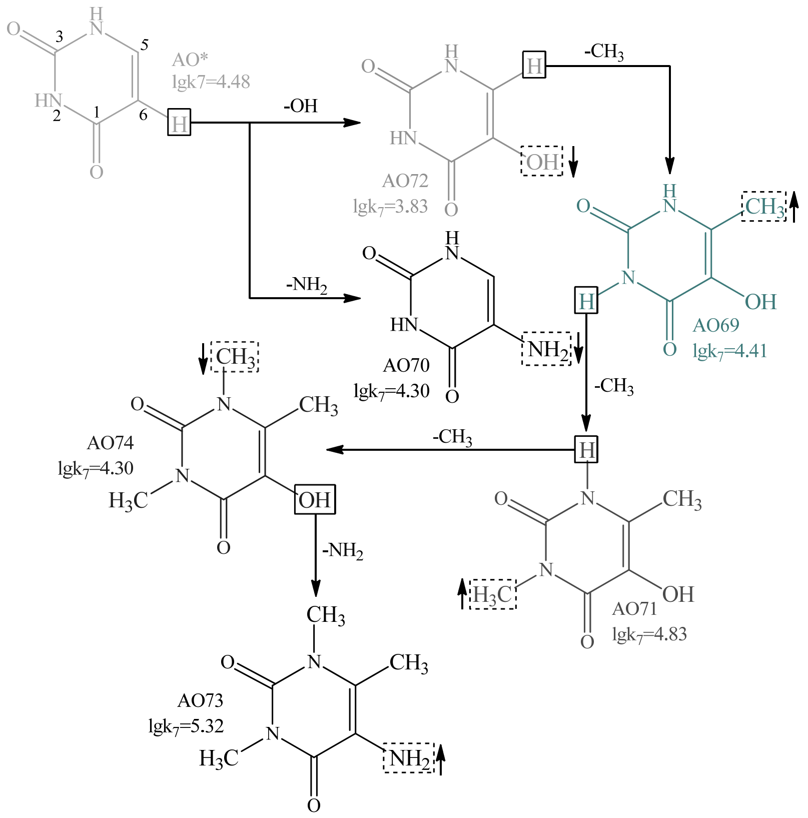

Figure S6. The effect of structural features on the antioxidant activity of compounds V. AO* is presented for comparison of lgk_7 values.

Replacing the H atom by OH in position R_3 decreases AOA of componds V (Figure 1). A similar effect is observed in the case of introducing the amino group. At the same time, introducing one or two methyl groups in R_2 and R_4 with the presence of OH in R_3 enhances

AOA. The next introduction of methyl in R_1 with the presence of two methyl groups in R_2 and R_4 and OH in R_3 decreases lgk_7. At the same time, in the case of amino group instead of OH in R_3, the effect is opposite.

Table S8. Electronic effects of meta and para substituents on the lgk_7 values of compounds I.

Code	Ri	σ_p	σ_m	F	R	lgk_7^{obs}
AO5	-H	0.00	–	0.03	0.00	3.60
AO8	$-CH_3$	-0.17	–	0.01	-0.18	4.08
AO9	$-COOCH_3$	0.45	–	0.34	0.11	2.96
AO5	-H	–	0.00	0.03	0.00	3.60
AO3	$-CH_3$	–	-0.07	0.01	-0.18	3.70
AO2	-H	0.00	–	0.03	0.00	4.64
AO7	$-NHC_6H_5$	-0.56	–	0.22	-0.78	6.23
AO10	$-OCH_3$	-0.27	–	0.29	-0.56	5.3
AO11	$-NO_2$	0.78	–	0.65	0.13	3.78
AO63	-OH	-0.37	–	0.33	-0.7	6.00
AO2	-H	–	0	0.03	0.00	4.64
AO4	-Cl	–	0.37	0.42	-0.19	4.26

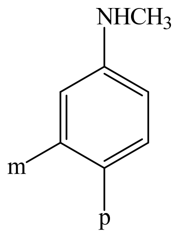

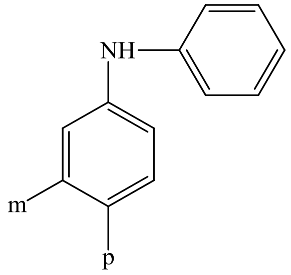

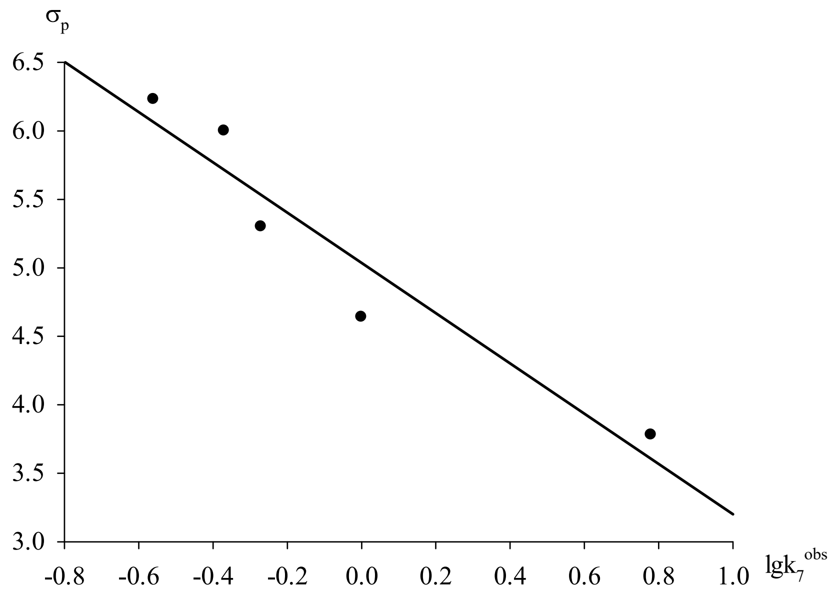

Figure S7. Effect of para-substituents on the lgk_7 values of compounds I.

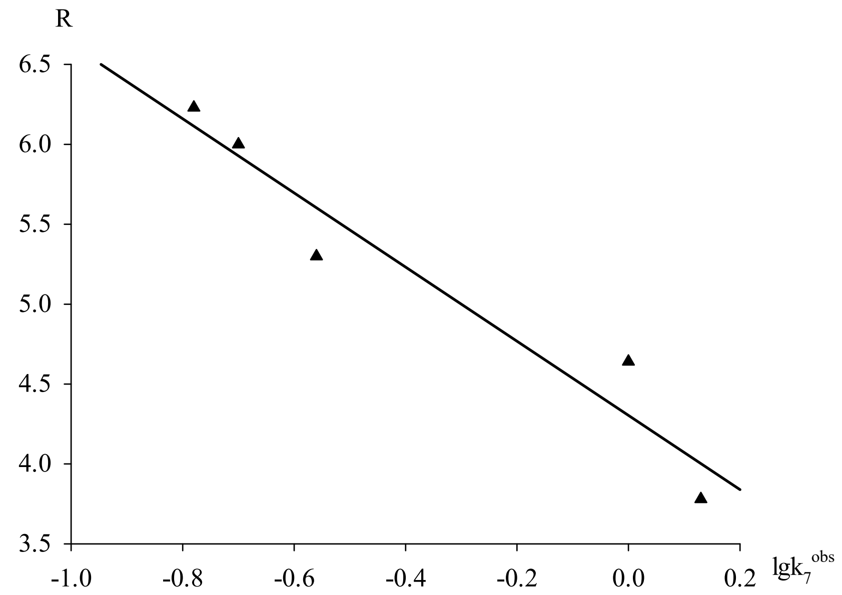

Figure S8. Inductive effect on the lgk_7 values of compounds I.

Table S9. Electron effects of meta and, para substituents on the lgk_7 values.

Code	Ri	σ_p	σ_m	F	R	lgk_7^{obs}
AO2	-H	0.00	–	0.00	0.00	3.48
AO25	-Cl	0.23	–	0.42	-0.19	3.67
AO22	$-CH_3$	-0.17	–	0.01	-0.18	4.30
AO21	$-OCH_3$	-0.27	–	0.29	-0.56	4.68
AO61	$-C_6H_{11}$	-0.15	–	0.03	-0.18	4.58
AO23	$-C(CH_3)_2C_2H_5$	-0.18	–	0.03	-0.21	4.21
AO51	-CN	0.66	–	0.51	0.15	3.81
AO63	$-NHC_6H_5$	-0.56	–	0.22	-0.78	6.00
AO2	-H	–	0.00	0.00	0.00	3.48
AO25	-Cl	–	0.37	0.42	-0.19	4.09
AO22	$-CH_3$	–	-0.07	0.01	-0.18	4.38
AO21	$-OCH_3$	–	0.12	0.29	-0.56	3.76

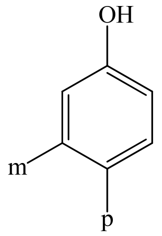

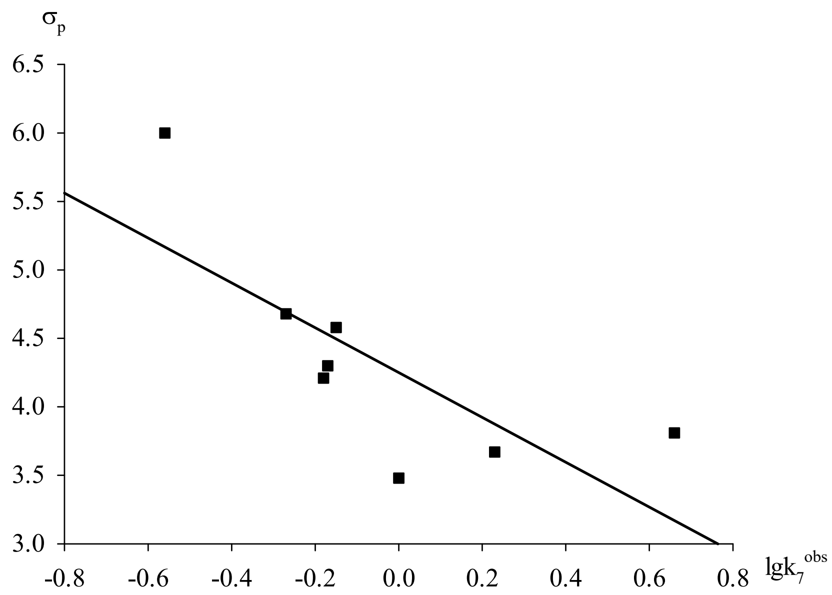

Figure S9. Effect of para-substituents on the lgk_7 values of compounds III.

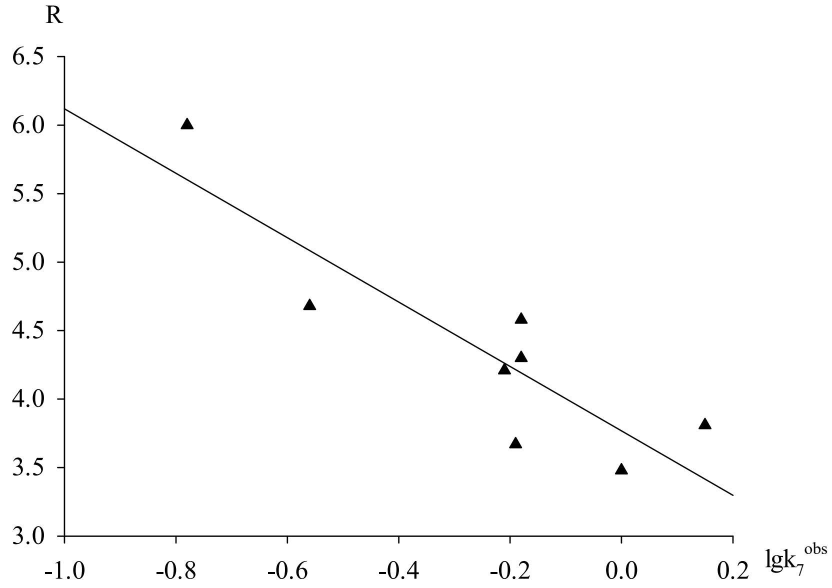

Figure S10. Inductive effect on the lgk_7 values of compounds III.

.
